# Type 3 inflammation–specific keratinocyte glutaminolysis promotes skin inflammation

**DOI:** 10.1172/jci.insight.203626

**Published:** 2026-09-08

**Authors:** Yongfei Hu, Hai Yu, Kaiyu Liang, Liyan Yuan, Wenjun Zhang, Zhe Zhuang, Suyun Ji, Xichun Xia

**Affiliations:** 1 Institute of Dermatology and Venereology, Dermatology Hospital, Southern Medical University, Guangzhou, China.; 2 Department of Dermatology, The First Affiliated Hospital of Jinan University & Jinan University Institute of Dermatology, Guangzhou, China.

**Keywords:** Autoimmunity, Dermatology, Metabolism, Skin, Therapeutics

## Abstract

Inflammatory cytokines reprogram keratinocyte metabolism, but the metabolic pathways that couple immune signals to pathological epidermal growth remain incompletely defined. Here, we identify glutaminase 1–mediated (GLS1-mediated) glutaminolysis as a metabolic program preferentially induced in keratinocytes under type 3 inflammatory conditions. Integrated transcriptomic, metabolomic, genetic, and functional analyses showed that IL-17A induced GLS1 expression and glutaminolysis in keratinocytes. Keratinocyte-specific *Gls1* deletion reduced the intracellular availability of arginine, proline, and methionine, impaired amino acid–dependent mTORC1 activation, disrupted redox homeostasis, and limited keratinocyte proliferation. Amino acid or antioxidant supplementation partially rescued these defects, whereas rapamycin blocked the amino acid–mediated proliferative rescue. *Gls1* deletion did not impair steady-state skin development or homeostasis and did not alter MC903-induced type 2 dermatitis, but it delayed wound re-epithelialization and attenuated imiquimod-induced psoriasiform inflammation. Loss of keratinocyte GLS1 also reduced epidermal chemokine expression and the accumulation of neutrophils and IL-17A–producing γδ T cells, revealing a role for glutaminolysis in amplifying epithelial-immune crosstalk. These findings define GLS1-mediated glutaminolysis as a context-specific metabolic checkpoint linking type 3 inflammation to keratinocyte proliferation and cutaneous immune amplification, and support locally or temporally controlled GLS1 inhibition as a potential therapeutic strategy for psoriasis.

## Abstract 2

A glutamine-fueled metabolic pathway helps skin cells overgrow and amplify psoriasis inflammation, suggesting a potential treatment target.

## Introduction

Programmed and continuous renewal of the skin epidermis is essential for maintaining skin homeostasis and preventing the entry of environmental pathogens. Epidermal integrity depends on a tightly regulated balance between proliferation and differentiation of keratinocytes derived from basal stem cells (1). Increasing evidence indicates that cellular metabolism is a crucial determinant of these processes and enables keratinocytes to adapt to changes in their local environment (2, 3). Skin injury caused by trauma, ultraviolet radiation, or infection activates resident immune cells and induces the production of inflammatory cytokines (4). Among the major immune programs, type 1 immunity primarily targets intracellular pathogens, whereas type 2 immunity is associated with helminth defense and allergic inflammation. Type 3 immunity is a barrier-centered response mediated predominantly by IL-17A, IL-17F, and IL-22, which protect epithelial surfaces against extracellular bacteria and fungi by promoting neutrophil recruitment, antimicrobial peptide production, and tissue repair. However, excessive or persistent type 3 inflammatory cytokines directly stimulate keratinocyte proliferation and inflammatory gene expression (5, 6), thereby disrupting epidermal homeostasis and contributing to chronic inflammatory diseases such as psoriasis (7). Inflammatory cytokines can also induce distinct metabolic programs in keratinocytes, and such metabolic reprogramming contributes to the pathogenesis of inflammatory skin diseases (8, 9). Nevertheless, the specific metabolic adaptations induced by type 3 cytokines and their functional roles in keratinocyte activation remain poorly defined. Elucidating these pathways is therefore important for understanding how type 3 inflammation perturbs epidermal homeostasis and for identifying new strategies to restore skin integrity.

Glutaminolysis is the process by which cells convert glutamine into TCA cycle metabolites through the activity of multiple enzymes (10). As an anaplerotic pathway, it is critical for providing intermediates that maintain mitochondrial metabolism, and contributes to generation of antioxidants. Apart from that, glutaminolysis participates in synthesis of nonessential amino acids, fatty acids, pyrimidines, and purines for cellular replication (11). Glutaminase 1 (GLS1) is a mitochondrial enzyme that serves as the very first step in the glutaminolysis pathway, which catalyzes the breakdown of glutamine to form glutamate. Pharmacological inhibition of GLS1 has emerged as a potential therapeutic strategy in some diseases. Recent studies have suggested a link between GLS1-mediated glutaminolysis and cutaneous inflammation (12, 13). GLS1 is expressed at low levels in homeostatic keratinocytes but is rapidly elevated, together with glutamate production, following IL-17A stimulation (13). Consistently, GLS1-mediated glutaminolysis is enhanced in keratinocytes from psoriatic lesions and wound-edge tissues (12, 13), both of which are characterized by type 3 inflammatory cytokine–driven keratinocyte hyperproliferation and re-epithelialization (6, 14, 15). These findings suggest that keratinocyte glutaminolysis is preferentially engaged in type 3 inflammatory microenvironments. However, whether it represents a type 3 inflammatory cytokine–induced metabolic program, and how keratinocyte-intrinsic glutaminolysis contributes to skin homeostasis, wound repair, and psoriasis remain incompletely defined.

In this study, we investigated whether keratinocyte-intrinsic glutaminolysis is preferentially engaged by type 3 inflammation and how this pathway regulates epidermal proliferation and cutaneous immune responses. We show that IL-17A induces GLS1-mediated glutaminolysis, which maintains amino acid–dependent mTORC1 activation and redox homeostasis in proliferating keratinocytes. Using keratinocyte-specific *Gls1*-deficient mice, we further distinguish its roles in steady-state skin, wound repair, type 2 dermatitis, and psoriasiform inflammation. Beyond supporting keratinocyte proliferation, GLS1-mediated glutaminolysis promotes epidermal chemokine expression and inflammatory cell recruitment, thereby amplifying epithelial-immune crosstalk during psoriasiform inflammation.

## Results

### Glutaminolysis is preferentially activated in keratinocytes under cutaneous type 3 inflammation.

To characterize glutaminolysis in keratinocytes during cutaneous type 3 inflammation, we analyzed the single-cell RNA expression profiles (GSE173706) of skin from patients with psoriasis and healthy controls. Integrated clustering identified 7 keratinocyte subsets: basal I–III, cycling, granular, spinous, and hair follicle keratinocytes, and their cell distributions in psoriatic and healthy control skin were summarized separately (Figure 1, A and B). Pathway analysis of genes upregulated in psoriatic basal subsets revealed enrichment of glutamine family amino acid metabolism, together with cytokine response, cell cycle, and IL-17 signaling pathways (Figure 1C). Consistently, AUCell analysis showed increased glutamate and glutamine metabolism scores in psoriatic keratinocytes, with the most consistent increases observed across the 3 basal subsets (Figure 1, D and E). Gene set enrichment analysis (GSEA) further confirmed enrichment of the glutamate metabolic process in psoriatic basal keratinocytes compared with both healthy basal keratinocytes and psoriatic spinous keratinocytes (Figure 1F). This transcriptional pattern was reproduced in an independent human psoriasis single-cell RNA-seq (scRNA-seq) dataset (GSE228421), in which glutamate metabolism was enriched among genes upregulated in psoriatic basal keratinocytes (Supplemental Figure 1, A and B; supplemental material available online with this article; https://doi.org/10.1172/jci.insight.203626DS1). Spatial transcriptomic analysis further showed increased expression of *GLS1*, *GLUL*, and *GOT2* in *KRT5*-enriched basal epidermal regions of psoriatic lesional skin compared with non-lesional skin (Supplemental Figure 1C). Consistent with the human data, scRNA-seq analysis of the imiquimod-induced (IMQ-induced) mouse model identified distinct basal, proliferative basal, spinous, and hair follicle keratinocyte subsets (Figure 1G and Supplemental Figure 1, D and E). Genes upregulated in IMQ-treated basal keratinocytes were enriched in glutamine metabolism, the TCA cycle, glutathione metabolism, nucleotide biosynthesis, and IL-17A signaling (Figure 1H). GSEA further demonstrated significant enrichment of the glutamine family amino acid metabolic process in IMQ-treated basal keratinocytes (Figure 1I), accompanied by increased expression of glutamine metabolism–related genes, including *Glul* and *Gclm* (Supplemental Figure 1F). To examine whether the activation of glutaminolysis is a disease-specific feature of psoriasis, we further analyzed 2 independent scRNA-seq datasets (GSE247047 and GSE222840) of atopic dermatitis (AD), which is considered a type 2 inflammatory microenvironment. In contrast with psoriasis, the top upregulated pathways were primarily associated with inflammatory responses, lipid metabolic processes, and oxidative phosphorylation in the AD basal keratinocytes (Supplemental Figure 2, A and B). Although GSEA revealed significant enrichment of mTORC1-mediated signaling in AD basal keratinocytes (Supplemental Figure 2C), no significant enrichment of glutaminolysis-related pathways was observed (Supplemental Figure 2C). The AUCell analysis across multiple epithelial clusters showed no significant induction of the “Glutamate and glutamine metabolism” gene set compared to healthy controls (Supplemental Figure 2, D and E). To test whether type 2 cytokines directly induce keratinocyte glutaminolysis, we analyzed air-liquid interface–cultured normal human epidermal keratinocytes (NHEK) cells from GSE282371 treated with IL-4/IL-13 with or without IL-17A. IL-4/IL-13 alone did not induce key glutaminolysis genes or enrich glutamine metabolism (Supplemental Figure 2, F and H), whereas the addition of IL-17A robustly upregulated these genes and enriched the glutamine family amino acid metabolic process (Supplemental Figure 2, G and I). Moreover, WT and K14-Cre *Gls1^fl/fl^* (cKO) mice showed comparable ear thickening, epidermal hyperplasia, serum TSLP and IgE elevation, and induction of *Tslp*, *Il33*, *Il25*, *Il13*, and *Il4* in AD-like dermatitis with MC903 (Supplemental Figure 3), indicating that keratinocyte GLS1 is dispensable for type 2 inflammation. These results indicate that glutaminolysis is not a general hallmark of inflammation but rather a cutaneous type 3 inflammatory microenvironment–specific metabolic adaptation in keratinocytes.

### IL-17A induces GLS1-mediated glutaminolysis to promote keratinocyte proliferation.

Given the enrichment of IL-17 signaling in psoriatic basal keratinocytes (Figure 1C and Supplemental Figure 1B), we examined whether IL-17A directly alters keratinocyte glutaminolysis. Untargeted metabolomic profiling revealed clear differences between vehicle- and IL-17A–treated primary mouse keratinocytes (Supplemental Figure 4, A and B). Based on enrichment analysis of Kyoto Encyclopedia of Genes and Genomes (KEGG) metabolic pathways related to significantly differential metabolites, amino acid metabolism was the most significant enrichment pathway (Figure 2A), which included upregulated glutamine, glutamic acid, arginine, glutathione, methionine, and proline (Figure 2B). The transcriptome profile showed 440 upregulated and 597 downregulated genes following IL-17A stimulation (Supplemental Figure 4C). Gene Ontology (GO) and KEGG analyses showed that these differentially expressed genes (DEGs) were associated with amino acid, glutathione, and related metabolic processes (Figure 2, C and D, and Supplemental Figure 4, D and E). GSEA further demonstrated positive enrichment of the glutamine metabolic process in IL-17A–treated keratinocytes (Figure 2E). In parallel, IL-17A increased GLS1 expression and intracellular glutamate levels in primary mouse keratinocytes (Figure 2F). These results demonstrated that GLS1-mediated glutaminolysis is an IL-17–induced metabolic program in keratinocytes. Corresponding to this metabolic rewiring, the proliferation was enhanced in IL-17A–treated keratinocytes, indicated by the most upregulated genes clustered in gene sets related to cell cycle (Figure 2G). However, the proliferation was markedly impaired in *Gls1*-deficient primary keratinocytes treated with IL-17A, but not with vehicle, on the basis of crystal violet staining and CCK-8 assay (Figure 2H). Similar results were also found in human keratinocytes (Figure 2I). All data implicated the critical role of GLS1-mediated glutaminolysis in promoting keratinocyte proliferation in the context of IL-17 stress.

### GLS1 maintains amino acid pools and redox homeostasis to support mTORC1 activation and keratinocyte proliferation.

To investigate the mechanisms underlying impaired proliferation in *Gls1*-deficient keratinocytes, the metabolome and transcriptome were profiled in IL-17A–treated mouse primary keratinocytes from cKO mice and their WT littermates. Metabolomic analysis revealed marked differences between the 2 genotypes (Supplemental Figure 5, A and B). Differential metabolites were significantly enriched in nucleotides and amino acid metabolism–related pathways (Figure 3A), with intracellular levels of glutamic acid, arginine, proline, methionine, and glutathione significantly downregulated in *Gls1*-deficient keratinocytes (Figure 3B). RNA-seq identified 1,327 upregulated and 1,199 downregulated genes in *Gls1*-deficient keratinocytes (Supplemental Figure 5C). Functional analyses associated these genes with glutamine, arginine and proline, cysteine and methionine, and glutathione metabolism (Supplemental Figure 5, D–G). Notably, these metabolic pathways and the amino acid–regulated mTORC1 gene set were negatively correlated in *Gls1*-deficient keratinocytes (Figure 3, C and D), indicating that *Gls1* ablation not only blocked glutamine metabolism, it also reduced the intracellular amino acid pools. Supplementation with a mixture of arginine, proline, and methionine partially rescued mTORC1 activation (Figure 3E) and keratinocyte proliferation (Figure 3F). Rapamycin abolished both effects (Figure 3, G and H), demonstrating that the proliferative rescue requires mTORC1 activation. Furthermore, *Gls1*-deficient keratinocytes also showed enrichment of glutathione metabolism and increased expression of oxidative stress–responsive genes (Figure 3, I and J, and Supplemental Figure 5H), accompanied by elevated cellular ROS levels (Figure 3K). Supplementation with glutathione or *N*-acetyl-cysteine partially restored cell growth (Figure 3L). Together, these findings indicate that GLS1-mediated glutaminolysis maintains intracellular amino acid pools and redox balance, thereby supporting amino acid–dependent mTORC1 activation and keratinocyte proliferation under IL-17A stimulation.

### Keratinocyte-specific Gls1 deletion does not disrupt normal skin development or homeostasis.

Given the requirement for GLS1 during inflammation-associated keratinocyte proliferation, we next examined its role in steady-state skin. Epidermal GLS1 expression was efficiently depleted in cKO mice (Figure 4A). Nevertheless, cKO mice showed no overt gross or histological abnormalities at either 1 week or 2 months of age (Figure 4B and Supplemental Figure 6A). Epidermal thickness, dermal white adipose tissue thickness, and hair follicle number were comparable between WT and cKO mice (Figure 4C). The expression and localization of the differentiation markers keratin 10 (K10) and K14 were also unchanged in cKO skin at both ages (Figure 4, D and E, and Supplemental Figure 6, B and C). Similarly, epidermal adhesion genes were expressed at comparable levels in WT and cKO mice (Figure 4F and Supplemental Figure 6D), and no differences in body weight were observed (Supplemental Figure 6E). Toluidine blue exclusion and neonatal weight loss assays further revealed intact epidermal barrier function in cKO mice (Figure 4, G and H). Under steady-state conditions, *Gls1* deletion did not markedly alter the expression of redox homeostasis genes or basal mTORC1 signaling (Figure 4, I and J, and Supplemental Figure 6F). Cutaneous inflammatory cytokine expression and the abundance of resident neutrophils, macrophages, dendritic cells, and T cells were likewise comparable between the 2 genotypes (Supplemental Figure 6, G and H). Together, these findings indicate that keratinocyte-specific *Gls1* deletion does not cause overt defects in skin development, epidermal differentiation, barrier function, or immune homeostasis.

### GLS1-mediated glutaminolysis supports injury-induced keratinocyte proliferation and wound repair.

Keratinocytes also undergo rapid proliferation in response to wounds, which is characterized as a transitory type 3 inflammatory microenvironment. To understand the role of GLS1-mediated glutaminolysis in wound repair, we assessed the re-epithelialization of mice from splinted 4-mm excisional wounds after 2, 4, 6, and 8 days (Figure 5A). Compared with their WT littermates, healing in cKO mice was significantly impaired at 4, 6, and 8 days (Figure 5, B and C). Specifically, by day 6 and day 8, the WT mice had largely completed wound healing, whereas all the cKO mice still exhibited persistent epidermal defects (Figure 5, B and C). Keratinocyte proliferation and migration both contribute to wound healing. To address the role of proliferation in re-epithelialization, we assessed the histology of the wound edge. Wounds in cKO mice had significantly fewer Ki-67^+^ cells within 500 μm of the wound edge when compared with WT mice (Figure 5D). To study cell migration in *Gls1*-deficient keratinocytes, we analyzed keratinocyte migration in scratch assays. Keratinocytes from cKO mice showed significantly delayed scratch closure compared with WT littermates (Supplemental Figure 7A). As expected, given the type 3 inflammatory microenvironment around the wound edge, GLS1 expression was significantly increased in the wound edge compared with the non-wound epidermis of WT mice, while GLS1 was undetectable in both of these 2 sites of cKO mice (Figure 5E). As a consequence, GLS1-mediated glutaminolysis and the associated arginine, proline, and methionine levels were significantly lower in the wound edge of cKO mice compared with WT mice (Figure 5F). In agreement with the scarcity of amino acids, the excisional wounds of cKO mice also showed impaired activation of mTORC1 (Figure 5G), thereby leading to defects of proliferation and migration in vivo. Interestingly, GLS1 was significantly elevated in human biopsies from the wound edge of healthy volunteers at 3 days after wounding (Figure 5H), which was validated in an independent NCBI GEO dataset (GSE28914) (Supplemental Figure 7B). In summary, GLS1-mediated glutaminolysis was necessary for keratinocyte proliferation, migration, and recovery in excisional wounds.

### Keratinocyte-specific Gls1 deletion attenuates psoriasiform skin inflammation.

To investigate the function of keratinocyte-intrinsic ablation of *Gls1* in psoriasis development, we established psoriasiform hyperplasia in WT and cKO mice through 2 models: topical application of IMQ and intradermal injection of IL-23. Undoubtedly, WT mice treated with IMQ showed a typical psoriasiform development in both clinical and pathological characteristics, including disease severity and acanthosis (Figure 6, A–C). In contrast, the epidermis of cKO mice was markedly protected from IMQ-induced psoriasiform hyperplasia (Figure 6, A–C). A similar resistance phenotype of cKO mice was also found in IL-23–induced psoriasiform dermatitis models (Figure 6D). In agreement with the defect of proliferation in *Gls1*-deficient keratinocytes, the epidermis of cKO mice showed fewer Ki-67^+^ basal cells than that of WT mice (Figure 6E). Western blotting confirmed that the epidermis of cKO mice showed impaired mTORC1 activation (Figure 6F), which was consistent with decreased glutamate, arginine, proline, and methionine levels (Figure 6G). In addition to keratinocyte proliferation, we examined the effect of inhibiting keratinocyte-specific GLS1-mediated glutaminolysis on inflammation through flow cytometry. Compared with WT mice, the skin of cKO mice showed significant decreases in the proportion and number of neutrophils (CD11b^+^Ly6G^+^) and γδ T17 cells (IL-17^+^TCRγδ^+^), but not Th17 cells (IL-17^+^CD4^+^), infiltrating the skin after treatment with IMQ (Figure 6H). Similarly, the mRNA levels of several chemokines and cytokines (*Ccl2*, *Ccl5*, *Ccl20*, *Cxcl3*, *Cxcl8*, *Cxcl10*, *Il1b*, *Il17a*, and *Il17f*) that were upregulated in IMQ-induced psoriasiform dermatitis showed significant decreases in cKO mice (Figure 6, I and J), suggesting a disrupted chemotaxis to immune cells by epidermal cells and reduced inflammation. Thus, keratinocyte-specific *Gls1* deletion not only restrains epidermal proliferation but also reduces chemokine expression and the accumulation of neutrophils and γδ T17 cells, identifying keratinocyte glutaminolysis as a metabolic amplifier of psoriasiform inflammation.

## Discussion

Inflammatory cytokines can reprogram keratinocyte metabolism, but whether distinct immune environments engage specific metabolic programs remains unclear (16). Here, we identify GLS1-mediated glutaminolysis as a keratinocyte-intrinsic program preferentially activated during type 3 inflammation. Multiomics and functional analyses show that IL-17A induces GLS1-dependent glutaminolysis, which supports amino acid–dependent mTORC1 activation, redox homeostasis, and keratinocyte proliferation. Although dispensable for steady-state skin homeostasis, this pathway also promotes chemokine production and immune cell recruitment, thereby amplifying psoriasiform inflammation.

Unlike AD, basal keratinocytes in psoriasis exhibit enhanced glutaminolysis across independent datasets, highlighting divergent metabolic remodeling mechanisms underlying different inflammatory skin diseases (9, 17). While both diseases exhibit epidermal barrier disruption and inflammation, their immune polarizations differ — type 3 inflammation (IL-17A, IL-22) in psoriasis versus type 2 inflammation (IL-4, IL-13) in AD (18, 19). This distinction underscores that glutaminolysis in keratinocytes is not a universal response to inflammation but rather context dependent, reflecting the nature of the cytokine milieu. The current data demonstrate that activation of glutaminolysis is dependent on type 3 inflammatory cytokines, as IL-17A directly induced GLS1 expression and glutamate accumulation. Thus, glutaminolysis emerges as a type 3 inflammation–restricted metabolic circuit and represents a conserved metabolic reprogramming feature of psoriatic epidermis.

Although glycolysis is upregulated under IL-17A stimulation, contributing to enhanced cell proliferation (2, 20), only glutaminolysis required the presence of type 3 inflammatory cues for activation. In contrast, glycolysis could be activated in a cytokine-independent manner (2, 21), suggesting distinct upstream control mechanisms. Functionally, both pathways supported keratinocyte proliferation, yet they were not redundant; glutaminolysis loss could not be compensated by glycolysis, underscoring its irreplaceable role in amino acid anabolism and mitochondrial redox control. These observations align with emerging evidence that metabolic compartmentalization allows epithelial cells to fine-tune proliferative versus stress-adaptive programs in response to inflammation (22). Mechanistically, glutaminolysis-derived metabolites orchestrate 2 key regulatory axes, mTORC1 activation and oxidative stress control (23). We show that *Gls1* deficiency reduces the intracellular abundance and availability of arginine, proline, and methionine, which are essential for cytosolic mTORC1 activation. GLS1-derived glutamate may link glutamine catabolism to amino acid homeostasis and redox control. Glutamate provides carbon and nitrogen for transamination reactions and serves as a precursor for proline- and arginine-related metabolism. In contrast, methionine is an essential amino acid and therefore cannot be synthesized de novo in mammalian cells; its reduced abundance may instead reflect altered uptake, recycling, or nutrient-sensing feedback following *Gls1* deletion. Thus, the depletion of intracellular amino acid pools may result from both the direct interruption of glutamine catabolism and secondary growth-restrictive responses that suppress nutrient uptake and anabolic activity. Importantly, supplementation with these amino acids restored mTORC1 signaling and partially rescued proliferation, supporting a causal relationship between amino acid anabolism and proliferative signaling. This is consistent with prior evidence that mTORC1 integrates amino acid availability to control epidermal renewal (24). Concomitantly, glutamate sustains the production of glutathione, a key antioxidant buffer; its loss leads to ROS accumulation and growth arrest, which could be reversed by antioxidant supplementation (25). Together, these mechanisms may explain the impaired amino acid–dependent mTORC1 activation and increased oxidative stress observed in *Gls1*-deficient keratinocytes. Thus, glutaminolysis provides a dual safeguard, fueling anabolic signaling while maintaining redox balance. This integrated control distinguishes glutaminolysis from other nutrient pathways and explains its indispensability in keratinocyte hyperproliferation. The coupling of mTORC1 and oxidative homeostasis may also explain why GLS1 inhibition normalizes, rather than ablates, epidermal proliferation. In psoriasis, mTORC1 hyperactivation drives uncontrolled keratinocyte growth and inflammatory gene expression (26), while moderate mTORC1 activity is necessary for differentiation and barrier maintenance (27). By attenuating the glutaminolysis input, GLS1 inhibition selectively restrains pathological signaling without compromising physiological turnover — an essential consideration for therapeutic translation.

Strikingly, keratinocyte-specific deletion of *Gls1* had no impact on normal skin development or differentiation, highlighting a remarkable degree of metabolic plasticity under homeostasis (2, 28, 29). In contrast, during wound repair, a transient state of type 3 inflammation (6, 30), GLS1 expression was markedly induced, and *Gls1* deletion impaired re-epithelialization. Human wound-edge biopsies similarly displayed increased GLS1 transcripts, underscoring a conserved role for glutaminolysis in injury-associated keratinocyte proliferation. These findings extend previous observations that IL-17A and IL-22 promote wound closure by stimulating keratinocyte growth and migration (6, 31). Thus, both wound healing and psoriasis exploit the same type 3 cytokine/glutaminolysis axis, albeit in transient versus chronic contexts. The selective requirement of glutaminolysis in “activated” rather than “homeostatic” epidermis suggests a metabolic switch that is dormant during quiescence but mobilized upon inflammatory cues — a paradigm reminiscent of metabolic activation in immune cells. Beyond intrinsic proliferation, keratinocyte glutaminolysis orchestrates immune-epithelial communication. *Gls1* deletion reduced chemokine production (CCL20, CXCL10, CXCL3), thereby limiting neutrophil and γδ T17 infiltration. This indicates that glutaminolysis reinforces inflammatory circuits by enabling keratinocytes to attract IL-17–producing cells, forming a feed-forward metabolic-immune loop. Such loops amplify inflammation; IL-17A induces GLS1 expression, which enhances chemokine output, recruiting more IL-17–secreting cells. Disruption of this loop by GLS1 inhibition dampened both epidermal hyperplasia and immune infiltration, demonstrating that metabolic modulation can achieve selective immune desensitization without broad immunosuppression. Similar metabolically driven epithelial-immune feedbacks have been reported in intestinal and airway epithelia (32, 33), but we believe our study delineates the first keratinocyte-centric metabolic circuit that sustains type 3 inflammation in the skin.

Psoriatic lesions often relapse from histologically normal skin regions harboring “metabolically primed” keratinocytes that remain hypersensitive to IL-17 after treatment (34, 35). Our data suggest that such pathogenic memory may depend on sustained glutaminolysis capacity. By pharmacologically or genetically suppressing GLS1, keratinocytes become metabolically desensitized to IL-17, failing to reactivate the hyperproliferative program. This metabolic desensitization could thus underpin long-term remission and complement cytokine blockade therapies. Given that current biologics effectively neutralize IL-17/IL-23 but do not erase the cellular memory of inflammation (36, 37), targeting glutaminolysis may represent a next-generation strategy to prevent disease recurrence. Future longitudinal studies assessing GLS1 activity in posttreatment skin could clarify whether residual glutaminolysis activity predicts relapse risk.

Collectively, our findings identify glutaminolysis as a key link between type 3 inflammation, metabolic adaptation, and epithelial regeneration. Keratinocytes therefore act not only as cytokine responders but also as metabolic effectors that translate immune cues into proliferative programs. The identification of a type 3 inflammation–specific metabolic axis has several translational implications. Previous studies in cancer and pulmonary vascular disease support the druggability of GLS1 while highlighting the context-dependent effects of its inhibition. Our finding that keratinocyte-specific *Gls1* deletion suppresses pathological epidermal proliferation without disrupting steady-state skin homeostasis further supports GLS1 as a potential therapeutic target in psoriasis. However, because GLS1 also promotes wound re-epithelialization, localized or temporally controlled inhibition may be preferable to sustained systemic blockade. Since glutaminolysis also governs Th17 cell differentiation, targeting GLS1 in both keratinocytes and T cells may provide complementary suppression of the IL-17 inflammatory axis.

## Methods

Further information can be found in Supplemental Methods.

### Sex as a biological variable.

Both male and female participants and animals were included in this study. However, sex was not considered as a biological variable in the study design or statistical analyses, and no sex-stratified analyses were performed. Therefore, the study was not specifically designed or powered to assess sex-dependent differences, and potential sex-related effects on the observed findings remain to be investigated in future studies.

### Participants.

We recruited 7 healthy volunteers and collected skin biopsies from the wound edge and the adjacent intact skin on the third day after injury. All these samples were used for RNA isolation or paraffin sectioning.

### Mice.

*Gls1^fl/fl^* mice (WT) and K14-driven Cre-recombinase mice were obtained from Cyagen Biosciences Inc, and all mice were maintained on the C57BL/6J background. *Gls1^fl/fl^* mice were provided by Yunfei Gao (Jinan University). We crossed female *Gls1^fl/fl^* mice with male K14-Cre mice to generate mice lacking *Gls1* specifically in keratinocytes (K14-Cre *Gls1^fl/fl^*, cKO). All the mice were housed in the SPF animal facility at Jinan University.

### Animal models.

Pilot experiments validated that the sample sizes previously reported for studies on IMQ-induced psoriasiform hyperplasia and excisional wound splinting would have sufficient power. For the IMQ-induced psoriasiform hyperplasia model, 2-month-old mice underwent depilation on their back and received daily topical treatment with 62.5 mg of Aldara cream (5% IMQ) for 5 consecutive days. An objective scoring system, based on the clinical PASI score for patients with psoriasis, was employed to assess the condition of the back skin daily. The scoring system encompassed erythema, scaling, and thickening, which were individually rated on a scale of 0 to 4 in a fully blinded fashion. The total score was obtained by adding the 3 index scores (score of 0–12). The tissues were harvested typically after 5 days of treatment. For the IL-23–induced psoriasiform hyperplasia model, 2-month-old mice were intradermally injected with 500 ng of recombinant mouse IL-23, dissolved in 25 μL PBS, into one ear, while 25 μL of PBS was injected into the contralateral ear every other day for a total of 5 injections. Ears were collected for analysis on day 10. For the measurement of acanthosis, the epidermal area was outlined, and its pixel size was measured using the lasso tool in Adobe Photoshop CS4. The relative area of the epidermis was calculated using the following formula: area = pixels/(horizontal resolution × vertical resolution). For the excisional wound–splinting model, 2-month-old mice were shaved and chemically depilated. A 4-mm biopsy punch was used to excise the dorsal skin at the midline between the shoulder and neck until reaching the panniculus carnosus. A sterile circular silicone splint was then affixed to the skin using Krazy glue and secured with 5-0 Prolene sutures. A topical antibiotic (bacitracin, polymyxin B, and neomycin) ointment was applied, and wounds were dressed with Tegaderm transparent dressing daily. The wound size was measured at the indicated time points.

### Analysis of public scRNA-seq datasets.

Public scRNA-seq datasets for human psoriasis (GSE173706, GSE228421) and AD (GSE247047 and GSE222840) (38–41) were downloaded and analyzed according the workflow of the Seurat R package. Integration analysis between public scRNA-seq data and our mouse scRNA-seq data were performed within R v4.3.3 and with Seurat v4.3.0 (42). Briefly, the 2000 anchors were used to integrate all Seurat objects created above: (a) by finding the integration anchors (Seurat:FindIntegrationAnchors), (b) by integrating the objects (Seurat:IntegrateData), (c) using all.genes to scale the data and regress out the nCount_RNA, and (d) calculating the top 30 PCAs and using them for dimensional reduction and identifying cell clusters by using a resolution of 0.5 and 1. DEGs were identified using the Seurat:FindMarkers function through pairwise comparisons of each cell type against its corresponding control. Visualization of the scRNA-seq data was achieved via the integrated plotting functions in the Seurat and ggplot2 R packages.

### scRNA-seq analysis of mouse psoriasis-like models.

Quality control was conducted to ensure all samples analyzed contained highly consistent quality. To remove low-quality fragmented cells, we filtered the ratio of mitochondrial genes to a maximum of 0.2. Cells expressing a minimum of 500 genes were kept, and counts were normalized for each cell by the total expression, multiplied by 1 × 10^6^, and log-transformed. The scVI software was used to integrate all the samples and to reduce the dimensionality of data for unified clustering (43). The scVI model implements a flexible generative model based on variation inference, creating a low-dimensional data representation for downstream analyses. We trained the scVI model on the combined dataset, which resulted in a significantly reduced dimensionality, and after the batch effect correction; the cells of all 10 samples showed correct clustering into each main population. To identify the DEGs, we used the Seurat:FindMarkers function and compared each cell types to its corresponding control (42). For visualization of the scRNA-seq data, the visualization methods in Seurat and ggplot2 R package were implemented.

### Gene set activity scoring using AUCell.

To evaluate the activity of specific metabolic pathways at single-cell resolution, we employed the AUCell R package (44). A specialized gene set for “glutamate and glutamine metabolism” was curated from GSEA MSigDB databases (45). For each cell, genes were ranked based on their expression levels, and the area under the curve (AUC) was calculated to assess the enrichment of the gene set within the top-ranking expressed genes. The resulting AUC scores were projected onto UMAP plots to visualize spatial activity and compared across disease (psoriasis/AD) and healthy control groups using the Wilcoxon rank-sum test to determine statistical significance.

### GSEA.

Pathway enrichment analysis was carried out using the GSEA (46) pre-ranked method with GSEApy (https://github.com/zqfang/GSEApy) (47), which enables the analysis of up- and downregulated genes simultaneously. Ten thousand permutations were performed at an FDR cutoff value of 0.05 to determine enriched gene sets.

### Statistics.

The experiments for animal models were independently repeated at least twice, and other experiments were independently repeated at least 3 times. GraphPad Prism 8.0 was used for data analysis. Data are presented as mean ± SD. Comparisons between 2 groups were performed using a 2-tailed Student’s *t* test. Comparisons among more than 2 groups were performed using 1-way analysis of variance (ANOVA) followed by Tukey’s multiple-comparison test. For longitudinal data involving repeated measurements over time, statistical significance was assessed using 2-way repeated-measures ANOVA followed by Šídák’s multiple-comparison test. Correlation analyses were performed using Spearman’s rank correlation test for non–normally distributed data. A *P* value of less than 0.05 was considered statistically significant.

### Study approval.

All participants provided written informed consent. The study was approved by the Institutional Review Board of The First Affiliated Hospital of Jinan University (approval number KY-2021-006) and performed in accordance with the declaration of Helsinki Principles. All animal procedures were performed under protocols approved by the Jinan University Institutional Animal Care and Use Committee (IACUC, Approval No. 20191129-04). All efforts were made to follow the Replacement, Refinement and Reduction guidelines.

### Data availability.

The raw bulk RNA-seq and scRNA-seq data reported in this paper have been deposited in the Genome Sequence Archive in the National Genomics Data Center (48, 49), China National Center for Bioinformation/Beijing Institute of Genomics, Chinese Academy of Sciences (GSA: CRA033340, CRA033085) and are publicly accessible at https://ngdc.cncb.ac.cn/gsa/browse/CRA033340 and https://ngdc.cncb.ac.cn/gsa/browse/CRA033085 Metabolome data reported in this paper have been deposited in the OMIX, China National Center for Bioinformation/Beijing Institute of Genomics, Chinese Academy of Sciences (accession no. OMIX013008) that are publicly accessible at https://ngdc.cncb.ac.cn/omix/release/OMIX013008 All data supporting the findings of this study are available within the article and its supplemental material. Numerical source data for all graphs, including individual data points and values underlying reported means, are provided in the accompanying Supporting Data Values file, with separate, clearly labeled worksheets for each applicable figure panel.

## Author contributions

Conceptualization: SJ and XX. Sample collection: HY, LY, and YH. Data curation: YH and HY. Data analysis: YH, HY, and KL. Data interpretation and Investigation: YH, HY, and KL. Data visualization: YH, HY, and KL. Resources: WZ and ZZ. Funding acquisition: XX. Writing, original draft: YH, KL, and XX. Writing, reviewing and editing: YH, HY, KL, LY, WZ, ZZ, SJ, and XX. Supervision: SJ and XX. All authors read, revised, and approved the final manuscript. YH, HY, and KL contributed equally. All authors had complete access to the study’s data and had final responsibility for the decision to submit the paper for publication. YH, HY, and KL accessed and verified the data.

## Conflict of interest

The authors have declared that no conflict of interest exists.

## Funding support

The following organizations provided financial support:

- National Natural Science Foundation of China, grant nos. 82473514 and 82103722, awarded to XX.
- Guangdong Special Support Program (Young Talents), grant no. 2025TQ09A459, awarded to XX.
- Science and Technology Program of Guangzhou, grant no. 2025A04J3957, awarded to XX.

## Supplementary Material

Supplemental data

Unedited blot and gel images

Supporting data values

## Figures and Tables

**Figure 1:**
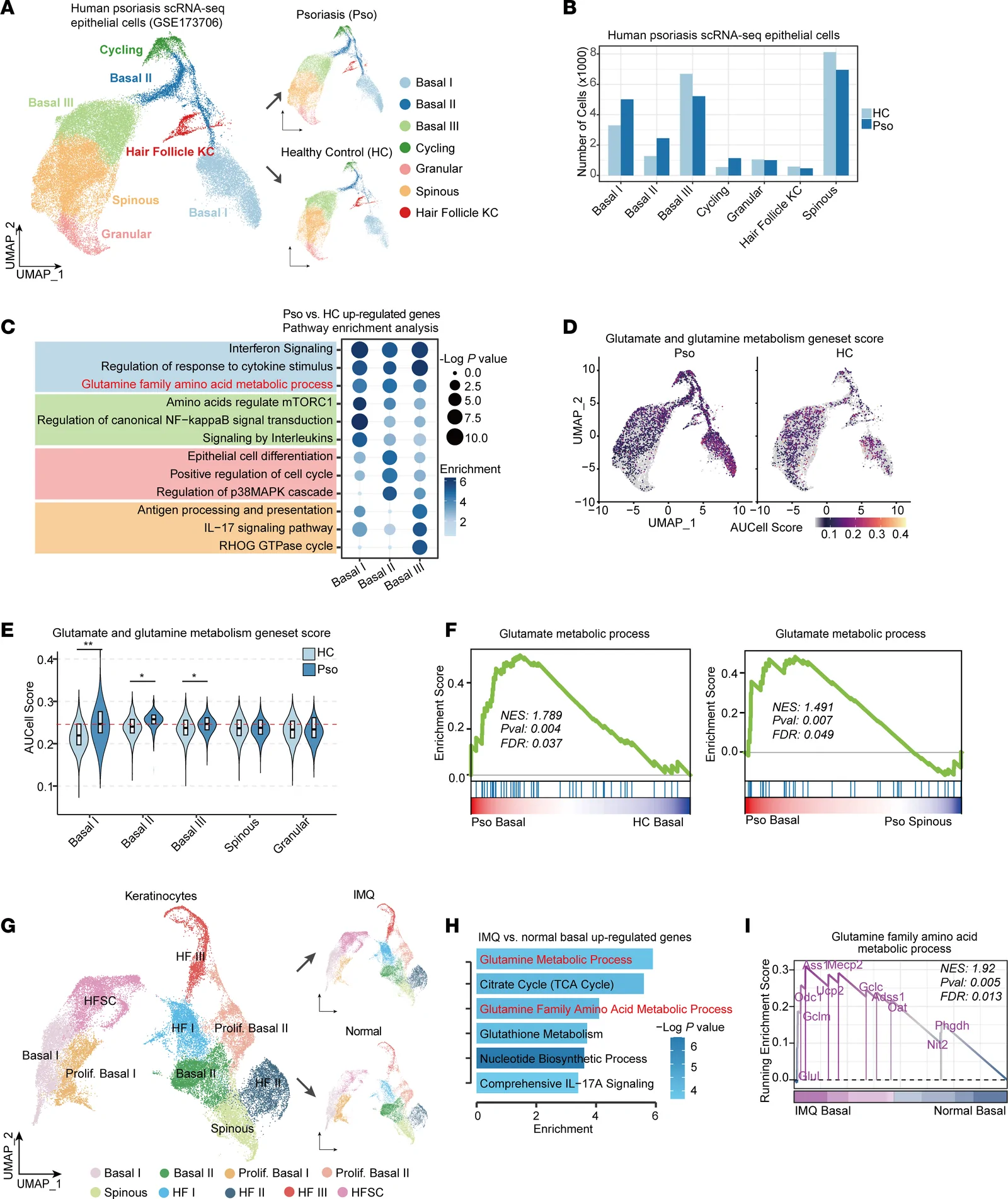
Glutaminolysis is preferentially activated in keratinocytes under cutaneous type 3 inflammation. (**A**) UMAP visualization of keratinocyte subclustering in the human psoriasis scRNA-seq dataset (GSE173706). Split UMAP plots illustrate the distribution of these cell types across psoriasis (Pso) and healthy control (HC) groups. (**B**) Bar chart illustrating the absolute cell numbers of various epithelial subpopulations in HC and Pso groups from dataset GSE173706. (**C**) Dot plot illustrating the functional enrichment of genes significantly upregulated in Pso versus HC across 3 distinct basal keratinocyte clusters. (**D**) UMAP dot plots showing the activity scores of the glutamate and glutamine metabolism gene set in Pso and HC groups. Scoring was quantified at single-cell resolution using the AUCell algorithm. (**E**) Violin plots depicting the distribution of AUCell-derived activity scores for the glutamate and glutamine metabolism gene set across various keratinocyte subpopulations. Statistical differences between Pso and HC within each cell type were evaluated using the Wilcoxon rank-sum test. *P < 0.05, **P < 0.01. (**F**) GSEA showing significant upregulation of glutaminolysis in psoriatic basal keratinocytes compared with HC (left panel), and in psoriatic basal versus spinous keratinocytes (right panel). (**G**) UMAP visualization of keratinocyte subclustering in the mouse psoriasis-like model scRNA-seq dataset (CRA033085), including the subdivision of the Basal cluster into Basal I and Basal II. Split UMAP plots depicting the distribution of keratinocyte subclusters in IMQ-treated and normal control groups, revealing comparable cell numbers for each subcluster across conditions. (**H**) Bar plot illustrating GO enrichment analysis of upregulated DEGs in Basal keratinocyte between IMQ and normal groups. (**I**) GSEA showing significant upregulation of glutaminolysis in IMQ-treated basal keratinocytes.

**Figure 2:**
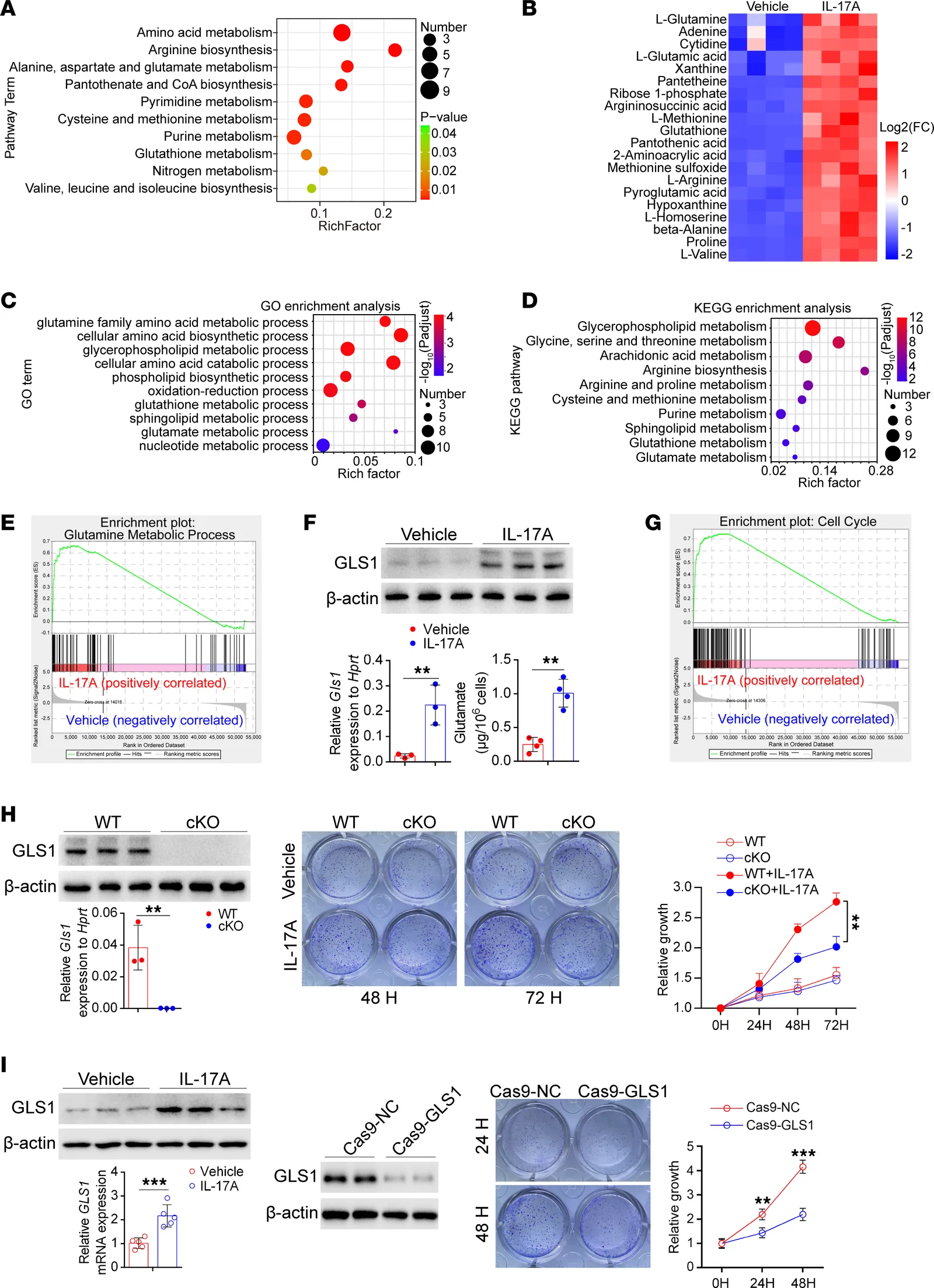
IL-17A induces GLS1-mediated glutaminolysis to promote keratinocyte proliferation. (**A** and **B**) LC-MS/MS analysis of differential metabolites in primary mouse basal keratinocytes treated with IL-17A versus vehicle (*n* = 4). Top 10 significantly enriched KEGG pathways of differential metabolites (**A**), and heatmap of top 20 significant differential metabolites (**B**) between IL-17A– and vehicle-treated keratinocytes. (**C**–**E**) Transcriptome analysis of DEGs in primary mouse basal keratinocytes treated with IL-17A versus vehicle (*n* = 3). Top 10 significantly enriched GO terms (**C**) and KEGG pathways (**D**), and GSEA of glutamine metabolic process (**E**) based on metabolism-related DEGs between IL-17A– and vehicle-treated keratinocytes. (**F**) Western blot analysis of GLS1 protein levels, RT-PCR measurement of *Gls1* mRNA, and intracellular glutamate levels in primary mouse basal keratinocytes treated with IL-17A versus vehicle (*n* = 3). (**G**) GSEA of cell cycle between IL-17A–and vehicle-treated primary mouse basal keratinocytes (*n* = 3). (**H**) Left: GLS1 protein and mRNA levels in primary basal keratinocytes from WT (*n* = 3) and cKO (*n* = 3) mice. Right: Growth rate of primary basal keratinocytes from WT (*n* = 3) and cKO (*n* = 3) mice, assessed by crystal violet staining and relative cell number after 48 hours and 72 hours of treatment with or without IL-17A. (**I**) Left: GLS1 protein and mRNA levels in NHEK cells treated with IL-17A versus vehicle. Middle: GLS1 protein in NHEK cells following CRISPR-mediated targeting of *GLS1*. Right: Growth rate of NHEK cells transduced with Cas9-GLS1 or Cas9-NC controls, assessed by crystal violet staining and relative cell number after 24 hours and 48 hours of treatment with IL-17A. Data are presented as mean ± SD and representative of 3 independent experiments with consistent results. Statistical significance was determined by 2-tailed Student’s *t* test (**F** and bar graphs in **H** and **I**) or 2-way repeated-measures ANOVA with Šídák’s multiple-comparison test (growth curves in **H** and **I**). **P < 0.01; ****P < 0.001.

**Figure 3:**
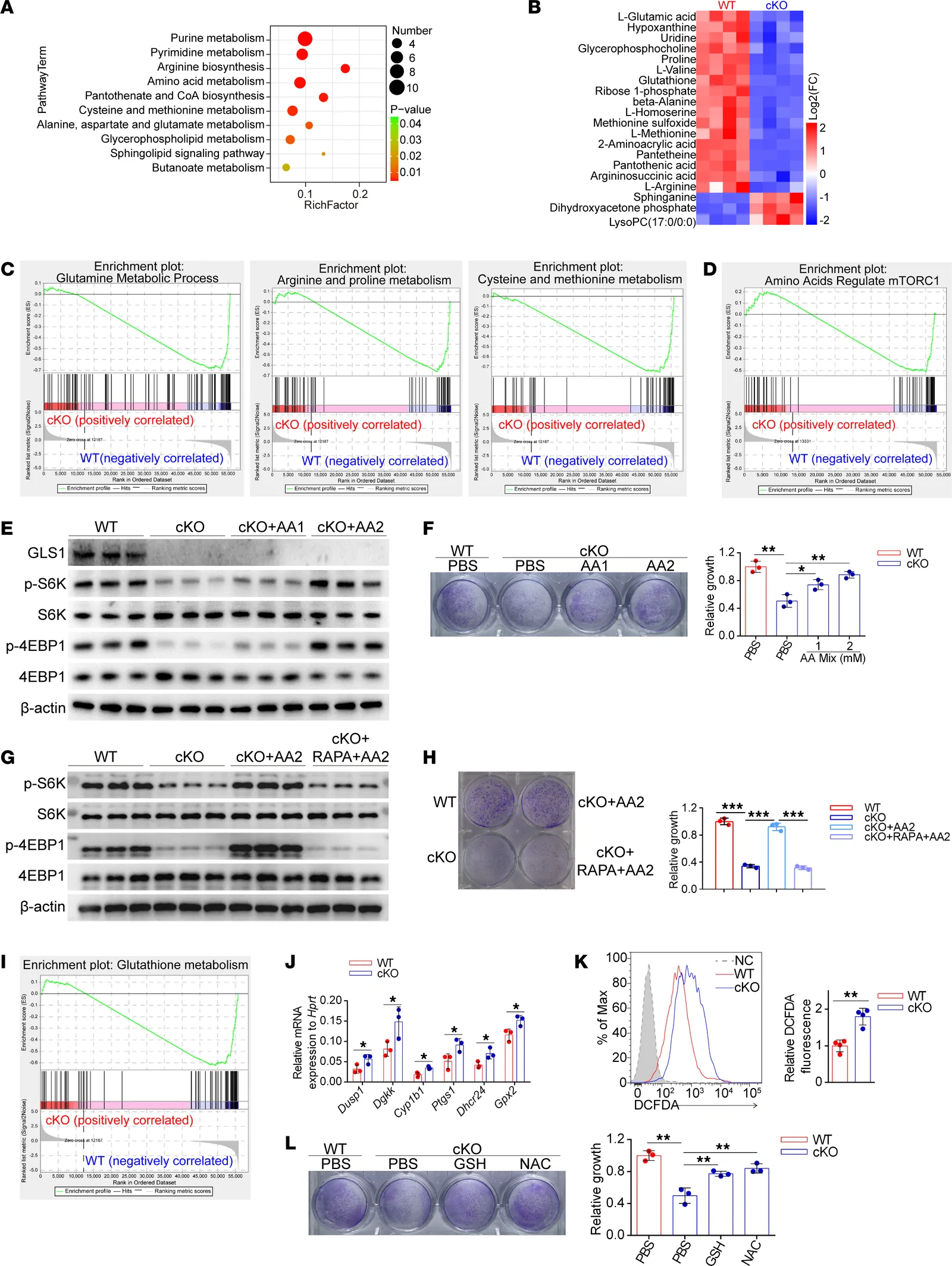
GLS1 maintains amino acid pools and redox homeostasis to support mTORC1 activation and keratinocyte proliferation. (**A** and **B**) LC-MS/MS analysis of differential metabolites in IL-17A–treated primary basal keratinocytes isolated from cKO versus WT mice (*n* = 4). Top 10 significantly enriched KEGG pathways of differential metabolites (**A**), and heatmap of top 20 significant differential metabolites (**B**) between WT and cKO keratinocytes. (**C** and **D**) Transcriptome analysis of DEGs in IL-17A–treated primary basal keratinocytes isolated from cKO versus WT mice (*n* = 3). GSEA of amino acid metabolism (**C**) and amino acids that regulate mTORC1 (**D**) based on metabolism-related DEGs between WT and cKO keratinocytes with IL-17A treatment. (**E** and **F**) Primary basal keratinocytes isolated from WT and cKO mice were treated with IL-17A and an amino acid mixture (arginine, proline, and methionine) rescue (AA1, 1 mM; AA2, 2 mM) or not (*n* = 3). Western blot for mTORC1 activation (**E**) and crystal violet staining for proliferation (**F**) of WT and cKO basal keratinocytes with and without amino acid supplementation. (**G** and **H**) Effects of rapamycin on amino acid–mediated restoration of mTORC1 signaling and cell growth. (**I**) GSEA of glutathione metabolism based on metabolism-related DEGs between WT and cKO basal keratinocytes with IL-17A treatment (*n* = 3). (**J**) RT-PCR measurement of oxidative stress–related genes in WT and cKO basal keratinocytes with IL-17A treatment (*n* = 3). (**K**) FACS analysis of the fluorescence after DCFDA staining of WT and cKO basal keratinocytes with IL-17A treatment (*n* = 4). (**L**) Crystal violet staining for proliferation (**F**) of WT and cKO basal keratinocytes with and without glutathione (GSH) or *N*-acetyl-cysteine (NAC) supplementation (*n* = 3). Data are presented as mean ± SD and representative of 3 independent experiments with consistent results. Statistical significance was determined by 1-way ANOVA with Tukey’s multiple-comparison test (**F**, **G**, and **L**) or 2-tailed Student’s *t* test (**J** and **K**). *P < 0.05; **P < 0.01; ***P < 0.001.

**Figure 4:**
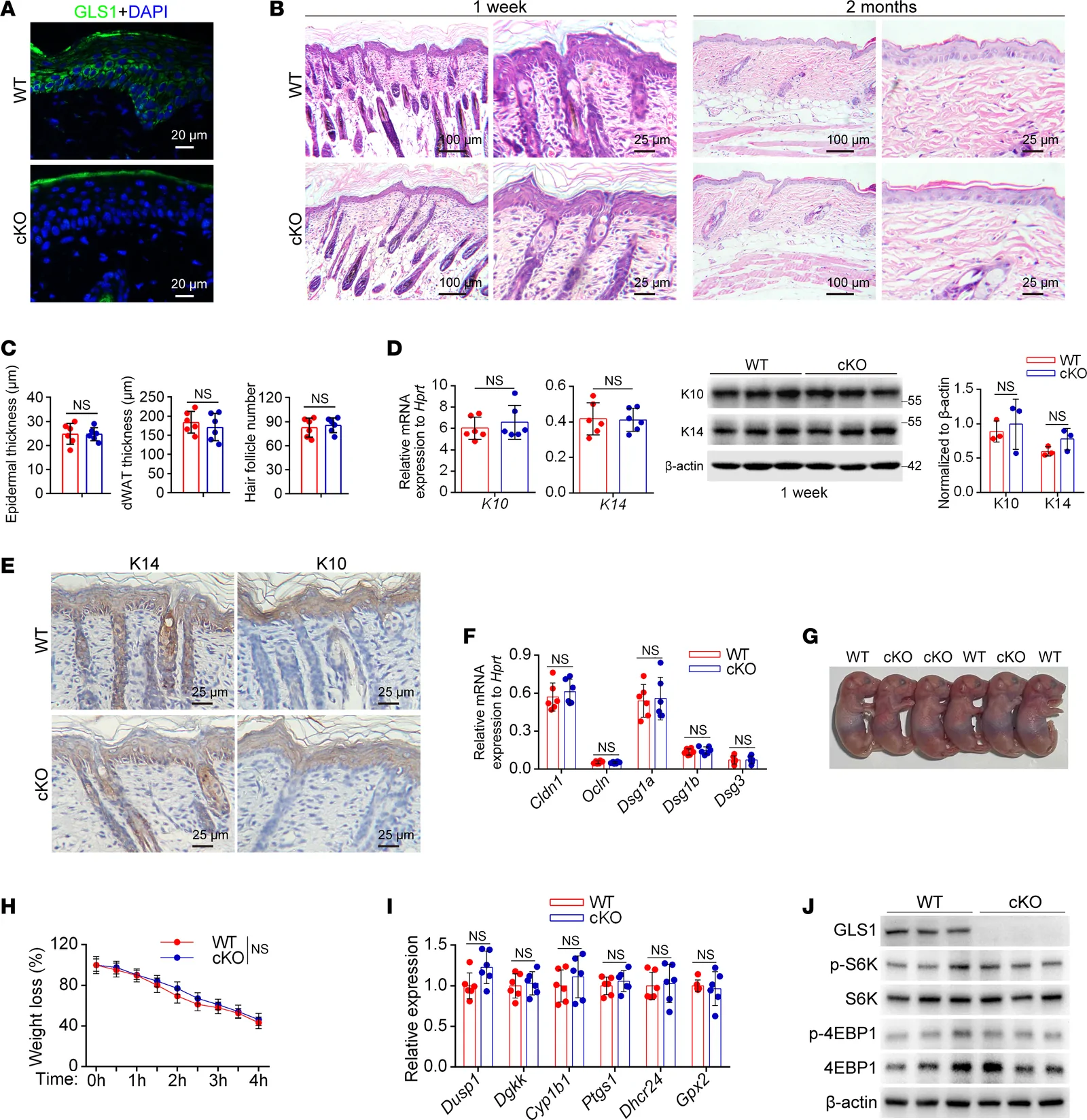
Keratinocyte-specific *Gls1* deletion does not disrupt normal skin development or homeostasis. (**A**) Immunostaining of GLS1 in skin from 1-week-old WT and cKO mice. GLS1 staining is absent in epidermis from cKO mice. (**B**) Histologic sections (H&E stained) of skin from 1-week- and 2-month-old WT and cKO mice show no histologic differences between the mice. (**C**) Quantification of epidermal thickness, dermal white adipose tissue (dWAT) thickness, and hair follicle number per 1-cm dorsal skin section in 2-month-old WT and cKO mice. (**D**) Left: Keratin 10 (K10) and K14 mRNA levels in harvested epidermis of 1-week-old WT (*n* = 6) and cKO (*n* = 6) mice. Right: K10 and K14 protein expression in the epidermis of 1-week-old WT (*n* = 3) and cKO (*n* = 3) mice. (**E**) Immunohistochemistry for K10 and K14 shows no differences in the respective suprabasal and basal expression for the epidermal keratins between 1-week-old WT and cKO mice. (**F**) RT-PCR measurement of adhesion genes in the epidermis of 1-week-old WT (*n* = 6) and cKO (*n* = 6) mice. (**G**) Toluidine blue staining of E18.5 embryos indicated no differences in epidermal permeability between WT (*n* = 3) and cKO (*n* = 3) mice. (**H**) Neonatal WT (*n* = 5) and cKO (*n* = 5) mouse littermates showed no differences in weight loss, suggesting transepidermal water loss was intact. (**I**) RT-PCR measurement of the indicated redox genes in basal keratinocytes of 1-week-old WT (*n* = 6) and cKO (*n* = 6) mice. (**J**) Western blot analysis of mTORC1 activation in basal keratinocytes of 1-week-old WT (*n* = 3) and cKO (*n* = 3) mice. Scale bars: 20 μm (**A**), 100 μm (**B** [low-magnification images]), and 25 μm (**B** [high-magnification images] and **E**). Data are presented as mean ± SD and representative of 2 independent experiments with consistent results. Statistical significance was determined by 2-tailed Student’s *t* test (**C**, **D**, **F**, and **I**) or 2-way repeated-measures ANOVA followed by Šídák’s multiple-comparison test (**H**).

**Figure 5:**
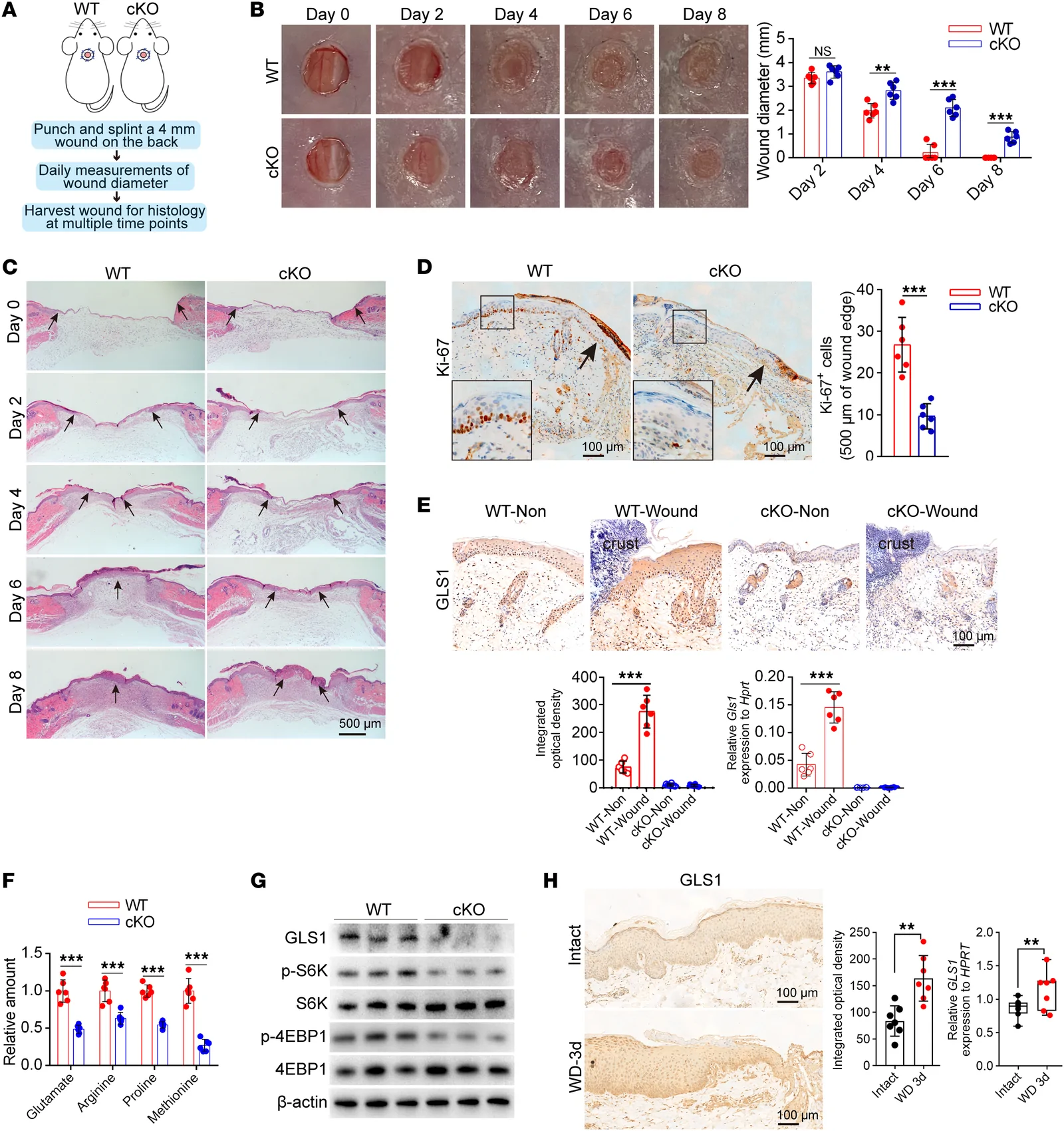
GLS1-mediated glutaminolysis supports injury-induced keratinocyte proliferation and wound repair. (**A**) Schematic for punch excision and wound splinting model. (**B**) Photo of splinted excisional wounds on the dorsal back and measurement of wound repair of WT and cKO mice on the indicated days after injury (*n* = 6). (**C**) H&E staining of dorsal skin wounds from WT and cKO mice on the indicated days after injury, assessing re-epithelialization (*n* = 6). (**D**) Ki-67 immunostaining of epidermis within 500 μm of the wound edge in WT and cKO mice on day 4 after injury (*n* = 6). (**E**) Immunohistochemical analysis and RT-PCR measurement of GLS1 expression in epidermis of wound edge and non-wounded skin from WT and cKO mice on day 4 after injury (*n* = 6). (**F**) LC-MS/MS analysis of amino acids in basal keratinocytes from the wound edge of WT and cKO mice on day 4 after injury (*n* = 6). (**G**) Western blot analysis of mTORC1 activation in basal keratinocytes from the wound edge of WT and cKO mice on day 4 after injury (*n* = 3). (**H**) Immunohistochemical analysis (**H**) and RT-PCR measurement of GLS1 expression in epidermis of human serial biopsies from the wound edge of healthy volunteers on day 3 after wounding (*n* = 7 individuals). Scale bars: 500 μm (**C**) and 100 μm (**D** and **H**). The high-magnification image in **D** was generated by applying a 2.5× digital zoom to the original image during image analysis. Data are presented as mean ± SD and representative of 2 independent experiments with consistent results. Statistical significance was determined by 2-way repeated-measures ANOVA followed by Šídák’s multiple-comparison test (**B**) or 2-tailed Student’s *t* test (**D**–**F**, **H**, and **I**). **P < 0.01; ***P < 0.001.

**Figure 6:**
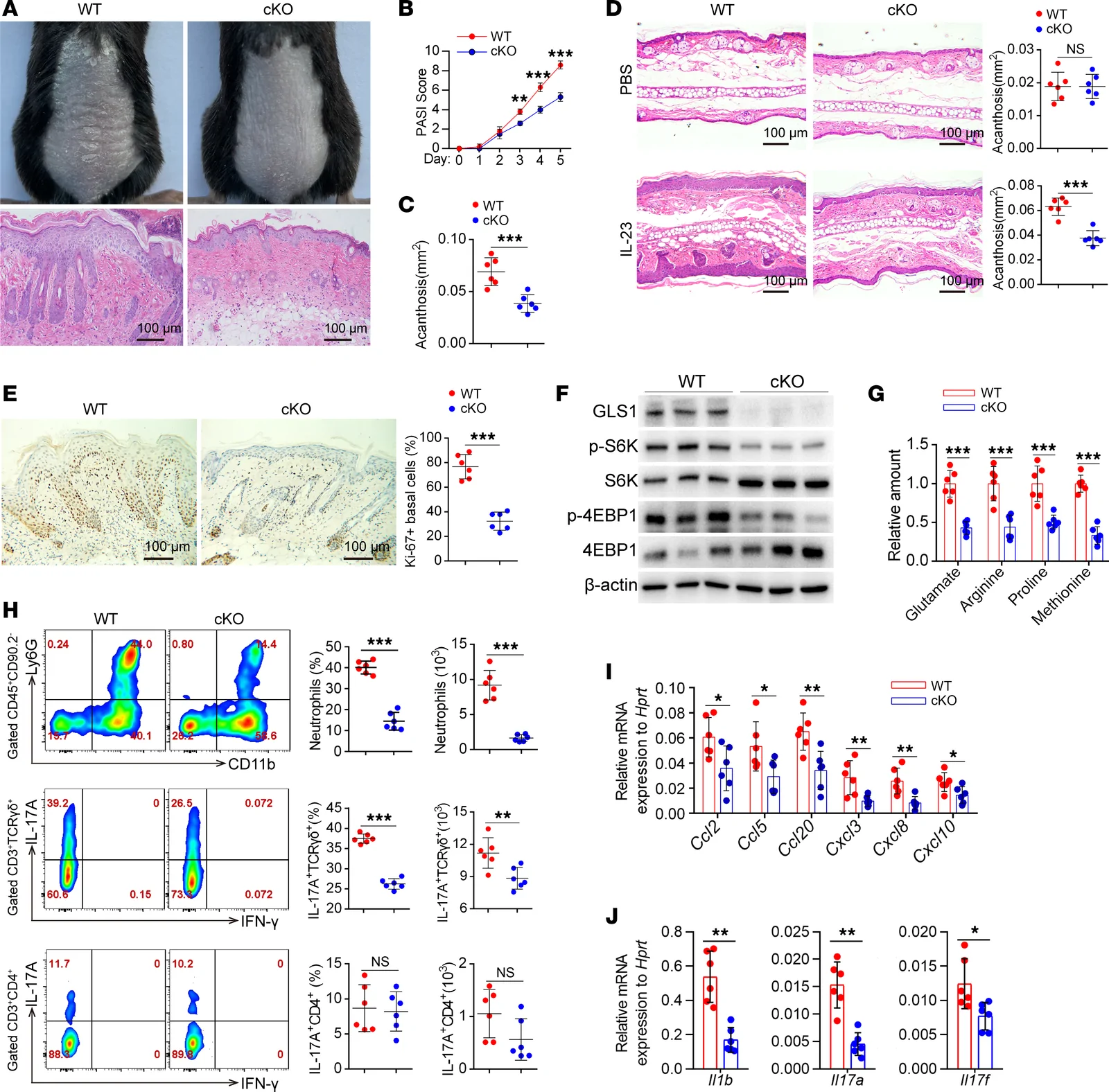
Keratinocyte-specific *Gls1* deletion attenuates psoriasiform skin inflammation. (**A**–**C**) WT and cKO mice were painted on their shaved back skin with IMQ-containing cream for 5 consecutive days (*n* = 6). Clinical manifestations and histologic sections (H&E staining) of skin lesions (**A**) (scale bars: 100 mm), PASI scores (**B**), and acanthosis (**C**) were recorded. (**D**) The ears of WT and KO mice were injected intradermally with PBS or IL-23 every other day 5 times (*n* = 6). Representative photographs of histologic sections (H&E staining) of the ears and acanthosis were measured. (**E**) Immunohistochemical analysis of Ki-67 expression in epidermis from WT and cKO mice in **A** (*n* = 6). (**F**) Western blot analysis of mTORC1 activation in basal keratinocytes from WT and cKO mice in **A** (*n* = 3). (**G**) LC-MS/MS analysis of amino acids in basal keratinocytes from WT and cKO mice in **A** (*n* = 6). (**H**) Flow cytometry and statistical analysis of the percentage and number of dermal neutrophils (gated on CD45^+^CD90.2^–^ lymphocytes), IL-17A^+^ γδ T cells (gated on CD3^+^TCRγδ^+^ cells), and IL-17A^+^CD4^+^ T cells (gated on CD3^+^CD4^+^ cells). (**I** and **J**) RT-PCR measurement of chemokine and cytokine expression in basal keratinocytes from WT and cKO mice in **A** (*n* = 6). Scale bars: 100 μm (**A**, **D**, and **E**) Data are presented as mean ± SD and representative of 2 independent experiments with consistent results. Statistical significance was determined by 2-way repeated-measures ANOVA followed by Šídák’s multiple-comparison test (**B**) or 2-tailed Student’s *t* test (**C**–**E** and **G**–**J**). *P < 0.05; **P < 0.01; ***P < 0.001.

## Version 1 09/08/2026

In-Press Preview

## Version 2 10/22/2026

Electronic publication
