# Supplementary material for: Type 3 inflammation–specific keratinocyte glutaminolysis promotes skin inflammation
Source: JCI Insight. 2026 Sep 8;11(20):e203626. doi: 10.1172/jci.insight.203626 (PMC13726564; doi:10.1172/jci.insight.203626)
Supplement: Supplementary file 1 [file jciinsight-11-203626-s113.pdf]

Supplementary Materials

Type 3 Inflammation-Specific Keratinocyte Glutaminolysis Promotes Skin Inflammation

Yongfei Hu[1#], Hai Yu[2#], Kaiyu Liang[1#], Liyan Yuan[1], Wenjun Zhang[1], Zhe Zhuang[1], Suyun Ji[1]*, Xichun Xia[1]*

Supplementary Methods

Supplementary Table S1. Primers used in this study.

Supplementary Figure S1. Single-cell RNA sequencing analysis of public human psoriasis and mouse psoriasis-like model dataset.

Supplementary Figure S2. Comparative Transcriptomic Analysis of Atopic Dermatitis Reveals Distinct Metabolic Profiles.

Supplementary Figure S3. Keratinocyte-specific Gls1 deletion does not alter MC903-induced AD-like skin inflammation.

Supplementary Figure S4. Metabolic and transcriptomic profiling of primary mouse basal keratinocytes treated with IL-17A.

Supplementary Figure S5. Metabolic and transcriptomic profiling of IL-17A-treated primary mouse basal keratinocytes from cKO versus WT mice.

Supplementary Figure S6. Skin development and homeostasis in WT and cKO mice.

Supplementary Figure S7. Migration of primary basal keratinocytes from WT and cKO mice, and GLS1 expression in human wound edge.

Supplementary Methods

Primary basal keratinocytes culture and treatment

Skin obtained from neonatal mice (post-natal day 0-3) was rinsed with sterile PBS (Invitrogen, 14190144) and placed into a 2 mL tube filled with 2 ml EpiLife Medium (Invitrogen, MEPI500CA) containing 4 mg/mL dispase (Invitrogen, 17105041). After incubating the skin tube for 14 h in a 4 °C refrigerator on a rotator, the dermis was peeled away from the epidermis. The separated epidermis was float on each drop of trypsin solution (Invitrogen, 12604021) in a dish, with the basal layer downward. The dish was incubated on a horizontal shaker at room temperature for 20 min, following the epidermis was vigorously rub back and forth to release single cells. Pipet the cell solution up-and-down gently to break any cell clumps, then the cell solution was filtered with a 100-μm cell strainer, centrifuged at 180 × g for 5 min, and resuspended in EpiLife medium supplemented with defined growth supplements (Invitrogen, S0125) and 100 U/ml antibiotics/antimycotics (Invitrogen, 15240062). The cells were counted and cultured in humidified incubator with 5% CO_2 at 37 °C, and fresh medium were replenished every other day. For treatment of the cells, IL-17A (200 ng/ml, Biolegend, 576006), GSH (Sigma, G6013), NAC (Sigma, A7250), Rapamycin (Sigma, 553210), and different concentrations of a mixture of amino acids, including arginine (Sigma, A5006), proline (Sigma, P0380) and methionine (Sigma, M9625), were supplemented in the EpiLife complete cell culture medium as indicated. Normal human epidermal keratinocytes (NHEKs) were obtained from the American Type Culture Collection (ATCC, PCS-200-011, USA) and cultured according to the manufacturer's instructions.

Histological Analyses and Immunoblotting

For immunohistochemistry (IHC), skin tissues were fixed in 4% paraformaldehyde and embedded in paraffin. Sections (5 μm) were deparaffinized, heat retrieved (incubated at 94-96 °C for 30 min in buffer containing 10 mM Tris and 1.0 mM EDTA, pH 8.0, then allowed to cool naturally), permeabilized (0.2% Triton X-100, 10 min), blocked in 3% BSA (Sigma, A9418), and then incubated with K10 (1:200, Proteintech, 18343-1-AP), K14 (1:200, Proteintech, 10143-1-AP), Ki-67 (1:400, Abcam, ab16667), GLS1 (1:500, Abcam, ab317032) primary antibodies. IHC and Hematoxylin (Vector, H3401)

and Eosin Y (Thermo, 6766007) staining (HE staining) were performed using standard protocols or under the manufacturer's instructions. Detection of IHC signal was performed with Vectastain Elite ABC kit (Vector Laboratories, Burlingame, CA) and DAB substrate kit for peroxidase (Vector Laboratories) followed by hematoxylin counterstaining (Vector Laboratories). For immunofluorescence of GLS1, after incubation with primary antibodies, slides were washed and incubated with Alexa Fluor 488 goat anti-rabbit secondary antibodies (Invitrogen, A-11008) at room temperature for 1 h, then washed and sealed with ProLong Gold antifade reagent with DAPI (Life Technologies, P36935). Images were acquired by Leica TCS SP5 confocal microscope system. For Western blotting, whole-cell extracts were separated on 8% or 12% SDS-PAGE gels, transferred to PVDF membranes (0.22 mm, Millipore) and probed with primary antibodies as follows: GLS1 (1:1000, CST, 88964; Abcam, ab317032), K10 (1:1,000, Proteintech, 18343-1-AP), K14 (1:1000, Proteintech, 10143-1-AP), p70 S6K (1:1000, CST, 9202S), Phospho-p70 S6K (Thr389) (1:1000, CST, 9205S), 4E-BP1 (1:1000, CST, 9644S), Phospho-4E-BP1 (Thr70) (1:1000, CST, 9455S), β-Actin (1:3000, Proteintech, 66009-1-Ig), and followed by secondary antibodies conjugated to HRP at a dilution of 1:3000 (CST, goat anti-rabbit, 98164S and goat anti-mouse, 91196S) and detection by the ECL System (Millipore, WBKLS0500). Protein content was quantified with a BCA protein assay kit (Thermo Pierce, 23227).

Cell proliferation Assay

For colony formation assay, an equal number of primary basal keratinocytes (2×10^4) were initially seeded into 24-well plates. At the specified time points, the cells were fixed and stained with 0.1% crystal violet solution (Sigma, C0775) for 30 minutes at room temperature. Subsequently, the cells were washed three times with ddH_2O, air-dried at room temperature, and finally photographed. For CCK-8 assay, primary keratinocytes (5×10^3) were seeded into 96-well plates for the indicated times. The cells were incubated for 3 h with 10 nM CCK-8 (MCE, HY-K0301), and the absorbance was measured at 450 nm.

In Vitro Migration Assay

Migration assay was conducted using wound healing assay. Primary basal keratinocytes (5×10^5) from WT or cKO mice were seeded into 6-well plates. After the cells have become a confluent cell monolayer, the mechanical scratching was used to make a cell-free gap in the monolayer. Then, the cell medium was replaced with fresh medium containing IL-17A (200 ng/ml) or not. The images of wound healing were acquired by using phase-contrast microscopy (Olympus) in 5 pre-determined fields over the indicated time. The average width of wound was used to quantify the rate of cell migration.

Toluidine blue staining

The newborn pups were dehydrated by series of 1 min-incubation in 25%, 50%, 75% methanol/PBS and 100% methanol, then rehydrated with the same series of methanol solutions and washed in PBS, followed by immersing in 0.1% (wt/vol) toluidine blue in PBS solution for 2 h. After staining, pups were rinsed with PBS and photographed using a digital camera.

Flow Cytometry

For cell surface staining, cells were incubated with specific antibodies for 15 min on ice in the presence of TruStain FcX (Biolegend, 101319, Clone: 93) to block FcγR binding. For intracellular staining, cells were stimulated with 50 ng/ml phorbol 12-myristate 13-acetate (Sigma, 524400) and 1 mg/ml ionomycin (Sigma, I9657) in the presence of GolgiStop (BD Biosciences, 554724) for 4 h. After stimulation, cells were fixed and permeabilized with BD Cytofix/Cytoperm™ Plus, followed by staining with fluorescent antibodies for an additional 30 minutes on ice in the dark. The following antibodies were used: APC-Cy7 anti-mouse CD45 (BD Biosciences, 557659, Clone: 30-F11), PerCP anti-mouse CD3 (Biolegend, 100326, Clone: 145-2C11), BD Horizon V500 Anti-Mouse CD90.2 (BD Biosciences, 561616, Clone: 30-H12), PE/Cy7 anti-mouse CD11b (Biolegend, 101215, Clone: M1/70), Brilliant Violet 421 anti-mouse CD11c (Biolegend, 117329, Clone: N418), APC anti-mouse Ly-6G (Biolegend, 108411, Clone: RB6-8C5), PE anti-mouse Ly-6C (Biolegend, 128007, Clone: HK1.4), FITC anti-mouse F4/80 (Biolegend, 123108, Clone: BM8), FITC anti-mouse MHCII (Biolegend, 107605, Clone: M5/114.15.2), PE/Cy7 anti-mouse IFN-γ (Biolegend,

505826, Clone: XMG1.2), APC anti-mouse IL-17A (Biolegend, 506916, Clone: TC11-18H10.1), Alexa Fluor® 488 anti-mouse TCR γ/δ (Biolegend, 118128, Clone: GL3), PE anti-mouse CD4 (Biolegend, 100408, Clone: GK1.5). For oxidative stress assay, cells were stained with 2 μM DCFDA (Invitrogen, D399) for 30 min, washed, trypsinized, collected on ice, centrifuged at 180 × g for 5 min, washed once with FACS buffer (Invitrogen, 00422226). All samples were acquired with FACS Verse flow cytometer (BD Biosciences) and analyzed with FlowJo software (TreeStar).

Quantitative Real-Time-PCR

Total RNA was isolated from cells or tissue using TRIzol reagent (Invitrogen, 15596026) and subjected to cDNA synthesis using PrimeScript™ II 1st Strand cDNA Synthesis Kit (Takara, 6110A). qRT-PCR was performed using SYBR Green qPCR Master Mix kit (Bimake, B21202) and CFX Connect Detection System (Bio-Rad). The expression of individual genes was calculated by a standard curve method and was normalized to the expression of *Hprt*. The primers used in qRT-PCR assays are shown in **Supplementary Table S1**.

Glutamate detection

Glutamate concentration was analyzed using a Glutamate Assay Kit (Sigma, MAK330) following the manufacturer's instructions. In general, 1×10^6 cells were homogenized in the Glutamate Assay Buffer followed by centrifugation. The supernatants or serum were deproteinized with a 10 kDa MWCO spin filter prior to addition to the reaction. Total 50 μl samples were separated into a 96-well plate and 100 μl of the appropriate Reaction Mix was added to each of the wells. After incubating the reaction for 30 minutes at 37 °C in dark, the absorbance was measured at 450 nm. All samples and standards were run in duplicate.

Metabolite profiling

Each sample was from one mouse, and four pairs of samples were used for metabolomic analysis. The harvested cells were snap frozen, and metabolites were extracted with 80% ice-cold methanol. Metabolite profiling was performed with LC–MS/MS. The peak area for each detected metabolite was normalized to the total ion count. The preprocessed datasets were mean-centered and unit-variance scaled. Principal

component analysis and hierarchical clustering of metabolites in different samples were performed in MetaboAnalyst 3.0.

RNA-sequencing analysis

Each sample was from one mouse, and three pairs of samples were used for for RNA-seq analysis. Primary basal keratinocytes were put into the Trizol solution (Sigma, T9424) for extraction of total RNA according to the manufacturer's instructions. RNA sequencing was performed by Shanghai Majorbio Bio-pharm Technology Co.,Ltd using an Illumina sequencer. The raw reads were aligned to the mm10 reference genome, using HiSat2 RNA-seq alignment software. The mapping rate was 90% overall across all the samples in the dataset. RSEM was used to quantify the gene expression counts from HiSat2 alignment files. Differential expression analysis was performed on the count data using R package DESeq2. P values obtained from multiple binomial tests were adjusted using false discovery rate with Benjamini and Hochberg method. Significant genes are defined by a fold-change of at least 2.0. The data were analyzed on the online platform of Majorbio Cloud Platform.

MC903-induced AD-like skin inflammation

Eight-week-old WT and cKO mice were topically treated on the ears with 1 nmol MC903 dissolved in ethanol or with ethanol alone once daily for 12 consecutive days. Body weight and ear thickness were recorded every 2 days throughout the experiment. Ear thickness was measured at the same anatomical location using a digital caliper. On day 12, mice were euthanized, and blood and ear tissues were collected. Serum TSLP and total IgE concentrations were measured by ELISA according to the manufacturers' instructions. Ear tissues were fixed in 4% paraformaldehyde, paraffin-embedded, sectioned, and stained with hematoxylin and eosin for histological evaluation. Additional ear tissue was used for RNA extraction and qRT-PCR analysis of type 2 inflammation-associated genes, including Tslp, *Il33*, *Il25*, *Il13*, and *Il4*.

Measurement of Epidermal, dWAT thickness and Hair follicles number

In full-thickness H&E-stained dorsal skin sections, epidermal thickness was measured as the perpendicular distance from the basement membrane to the outer boundary of the nucleated epidermis. dWAT thickness was measured as the perpendicular distance

between the lower boundary of the dermis and the upper boundary of the panniculus carnosus. Hair follicles were counted and normalized to the length of the overlying epidermis. Three nonadjacent sections separated by at least 100 μm were analyzed for each mouse, and the mean number of hair follicles per 1-cm of epidermal length was used as one biological replicate.

Supplementary Table S1

Primers used in this study.

Gene name	Forward Primer (5' to 3')	Reverse Primer (5' to 3')
Gls1	CTACAGGATTGCGAACATCTGAT	ACACCATCTGACGTTGTCTGA
Dusp1	GTTGTTGGATTGTCGCTCCTT	TTGGGCACGATATGCTCCAG
Dgkk	CCTGGCTCTACATCAGTAACTGC	AATTTGAGGAGTGACCCAACAG
Cyp1b1	CACCAGCCTTAGTGCAGACAG	GAGGACCACGGTTTCCGTTG
Ptgs1	ATGAGTCGAAGGAGTCTCTCG	GCACGGATAGTAACAACAGGGA
Dhcr24	CTCTGGGTGCGAGTGAAGG	TTCCCGGACCTGTTTCTGGAT
Gpx2	GCCTCAAGTATGTCCGACCTG	GGAGAACGGGTCATCATAAGGG
K10	CAGTTCTCTTCCTCCCGCAG	GTAACCACCTGATGAGCCCC
K14	TGGTATCGGTGGTGGCTCTA	GAAGCGAGAGGAGGTAACCG
Cldn1	TTTGGCCAGGCCCTCTTTAC	AGGTTGTTTTCCGGGGACAG
Ocln	CTCGGTACAGCAGCAATGGT	CATAGTGGTCAGGGTCCGTC
Dsg1a	TGGGGAATATAAAGGAACAGTGCT	CGTTGTGGGTTCTCAGTGGA
Dsg1b	TGGGGAATATAAAGGAACAGTGCTA	GTCACTGGGTCACGATTACCA
Dsg3	AGTACCGAGTGCAGTCAACG	CCCGTGTCCTCATCAGTAGC
Il1b	GAAATGCCACCTTTTGACAGTG	TGGATGCTCTCATCAGGACAG
Il17f	TGCTACTGTTGATGTTGGGAC	AATGCCCTGGTTTTGGTTGAA
Il17a	TTTAACTCCCTTGGCGCAAAA	CTTTCCCTCCGCATTGACAC
Ccl2	TTAAAAACCTGGATCGGAACCAA	GCATTAGCTTCAGATTTACGGGT
Ccl5	GCTGCTTTGCCTACCTCTCC	TCGAGTGACAAACACGACTGC
Ccl20	GCCTCTCGTACATACAGACGC	CCAGTTCTGCTTTGGATCAGC
Cxcl3	CACCCTACCAAGGGTTGATTTTG	GTGGCTATGACTTCTGTCTGGG
Cxcl8	TCAATGCCTGAAGACCCTGCCAA	TGGGTTCTTCCGTTGAGGGACAGC
Cxcl10	CCAAGTGCTGCCGTCATTTTC	GGCTCGCAGGGATGATTTCAA
Tslp	GCAAATCGAGGACTGTGAGAGC	TGAGGGCTTCTCTTGTTCTCCG
Il33	CTACTGCATGAGACTCCGTTCTG	AGAATCCCGTGGATAGGCAGAG
Il25	TGGCTGAAGTGGAGCTCTGCAT	CCCGATTCAAGTCCCTGTCCAA
Il13	AACGGCAGCATGGTATGGAGTG	TGGGTCCTGTAGATGGCATTGC
Il4	ATCATCGGCATTTTGAACGAGGTC	ACCTTGGAAGCCCTACAGACGA
Hprt	CCTAAGATGAGCGCAAGTTGAA	CCACAGGACTAGAACACCTGCTAA
GLS1	AGGGTCTGTTACCTAGCTTGG	ACGTTCGCAATCCTGTAGATTT
HPRT	CCTGGCGTCGTGATTAGTGAT	AGACGTTCAGTCCTGTCCATAA

Supplementary Figure S1.

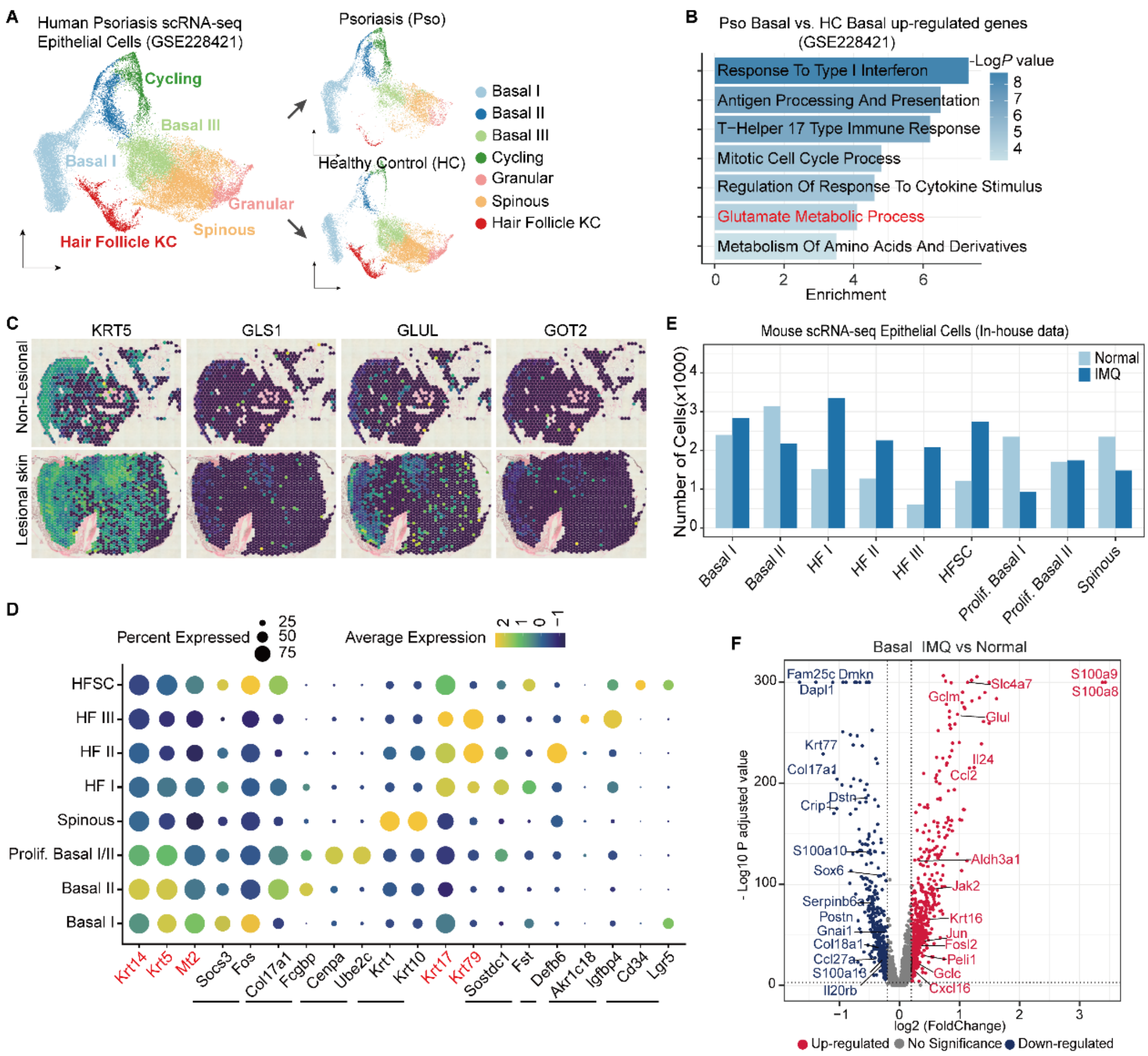

Supplementary Figure S1. Single-cell RNA sequencing analysis of public human psoriasis and mouse psoriasis-like model dataset. A. UMAP visualization of keratinocyte subclustering in the human psoriasis scRNA-seq dataset (GSE228421). Split UMAP plots illustrating the distribution of distinct keratinocyte cell types across psoriasis (Pso) and healthy control (HC) groups. **B.** Bar plot illustrating GO enrichment analysis of upregulated DEGs in Basal keratinocyte between Pso and HC from psoriasis dataset (GSE228421). **C.** Spatial transcriptomics analysis of glutaminolysis-related genes expression (*GLS1, GLUL, GOT2*) in basal keratinocytes between psoriatic lesional skin and non-lesional skin in the GSE202011 dataset. **D**. Dot plot illustrating the expression of marker genes across keratinocyte subclusters in mouse psoriasis-like models scRNA-seq dataset. **E.** Bar chart illustrating the absolute cell numbers of various epithelial subpopulations in IMQ and Normal groups from mouse scRNA-seq data (in-house data). **F.** Volcano plot of DEGs in the Basal subcluster between IMQ and Normal group, with each point representing a gene.

Supplementary Figure S2.

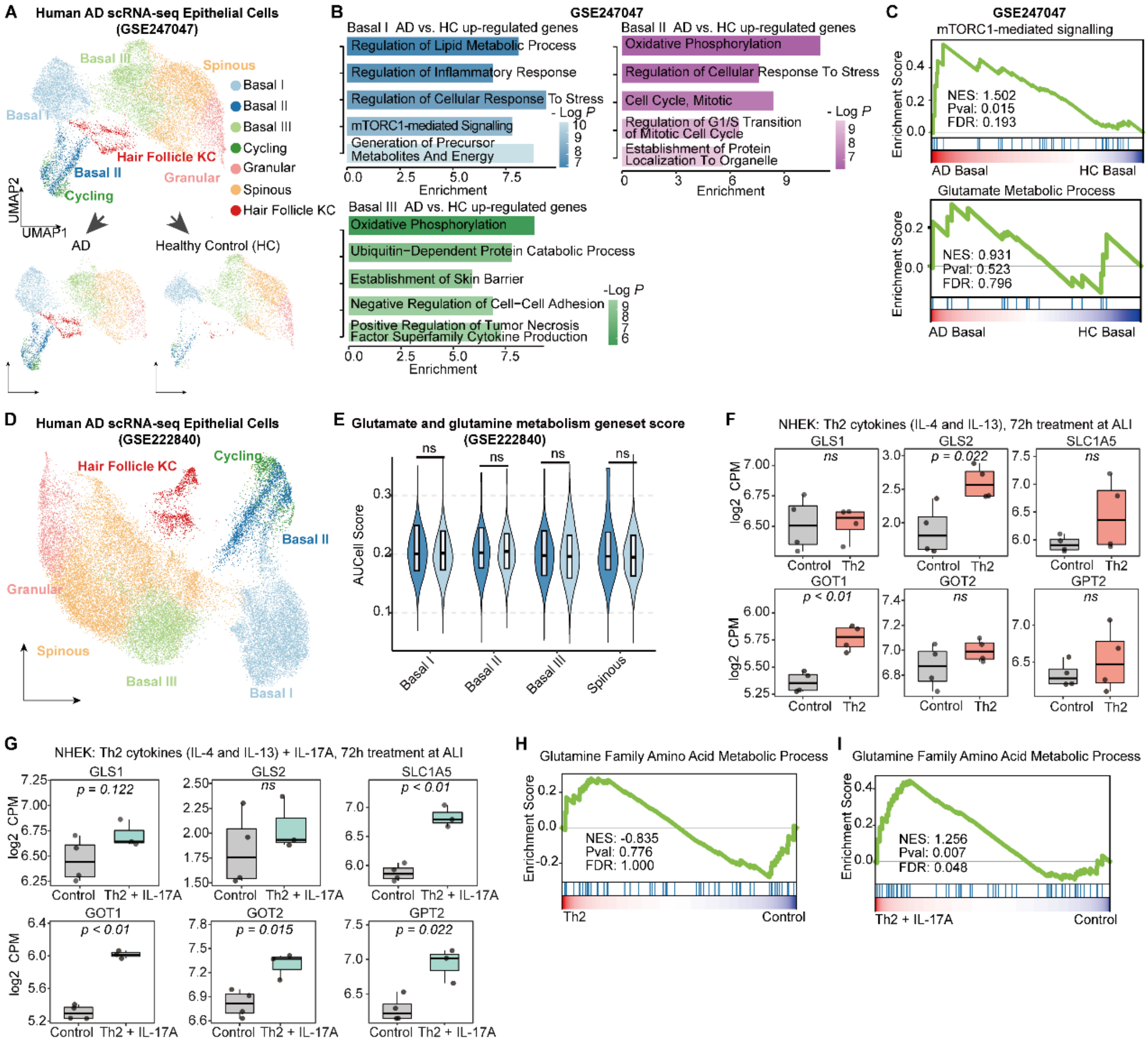

Supplementary Figure S2. Comparative Transcriptomic Analysis of Atopic Dermatitis Reveals Distinct Metabolic Profiles. A. UMAP visualization of epithelial cells from human atopic dermatitis (AD) lesions and healthy controls (HC) from the GSE247047 dataset. **B.** Pathway enrichment analysis of upregulated genes in AD versus HC for Basal I (blue), Basal II (purple), and Basal III (green) clusters. Dominant enriched pathways include lipid metabolic processes, inflammatory responses, and oxidative phosphorylation. **C.** GSEA enrichment of the "mTORC1-mediated signaling" and "Glutamate Metabolic process" gene set in Basal cells from AD patients compared to HC (GSE247047). **D.** UMAP projection of epithelial cell clusters from an independent AD dataset (GSE222840), validated to ensure the consistency of cell-type identification across different AD cohorts. **E.** AUCell activity scores for the glutamate and glutamine metabolism gene set in the GSE222840 dataset. Violin plots show the distribution of metabolic scores across Basal I, II, III, and Spinous cells, comparing AD (dark blue) and HC (light blue). ns: Wilcoxon rank-sum test not significant. **F.** Expression of glutamine metabolism-related genes in NHEK cells treated with Th2 cytokines. Box plots showing log2 counts per million (CPM) for GLS1, GLS2, SLC1A5, GOT1, GOT2, and GPT2 following 72h treatment with Th2 cytokines (IL-4 and IL-13) at the air-liquid interface (ALI) (GSE282371). t-test p-values are indicated; ns: not significant. **G.** Synergistic induction of metabolic genes by Th2 + IL-17A. mRNA expression levels of the metabolic gene panel in NHEK cells treated with a combination of Th2 cytokines and IL-17A. **H.** GSEA plot for "Glutamine Family Amino Acid Metabolic Process" in Th2-treated vs. Control cells. **I.** GSEA plot for the same metabolic pathway comparing Th2 + IL-17A treated cells vs. Control.

Supplementary Figure S3.

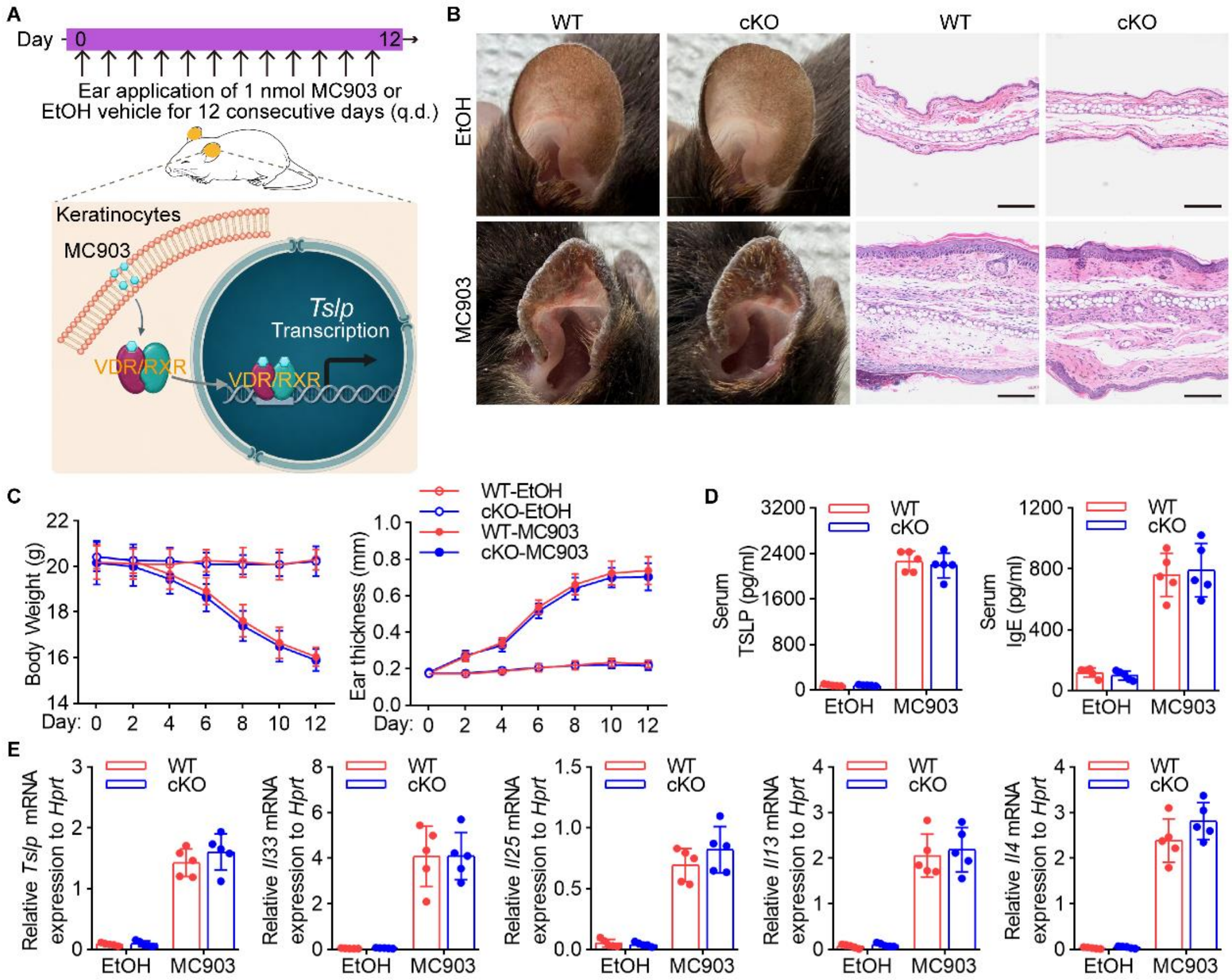

Supplementary Figure S3. **Keratinocyte-specific Gls1 deletion does not alter MC903-induced AD-like skin inflammation. A.** Schematic graph of the procedure of MC903-induced mouse model and its mechanism. **B.** Representative pictures and H&E staining of mouse ears (n = 5) on day 12. Scale bar, 100 μm. **C.** Measurement of body weight and ear thickness change in both WT and cKO mice (n = 5). **D.** Secreted TSLP and IgE levels measured by ELISA assay in serum from mice (n = 5) on day 12. **E.** Quantitative real-time PCR analysis of various cytokines levels in the ears (n = 5). Data are presented as mean ± SD and representative of two independent experiments with consistent results. Statistical significance was determined by two-way repeated-measures ANOVA with Šídák's multiple-comparisons test (**C**), and one-way ANOVA with Tukey's multiple-comparisons test (**D, E**).

Supplementary Figure S4.

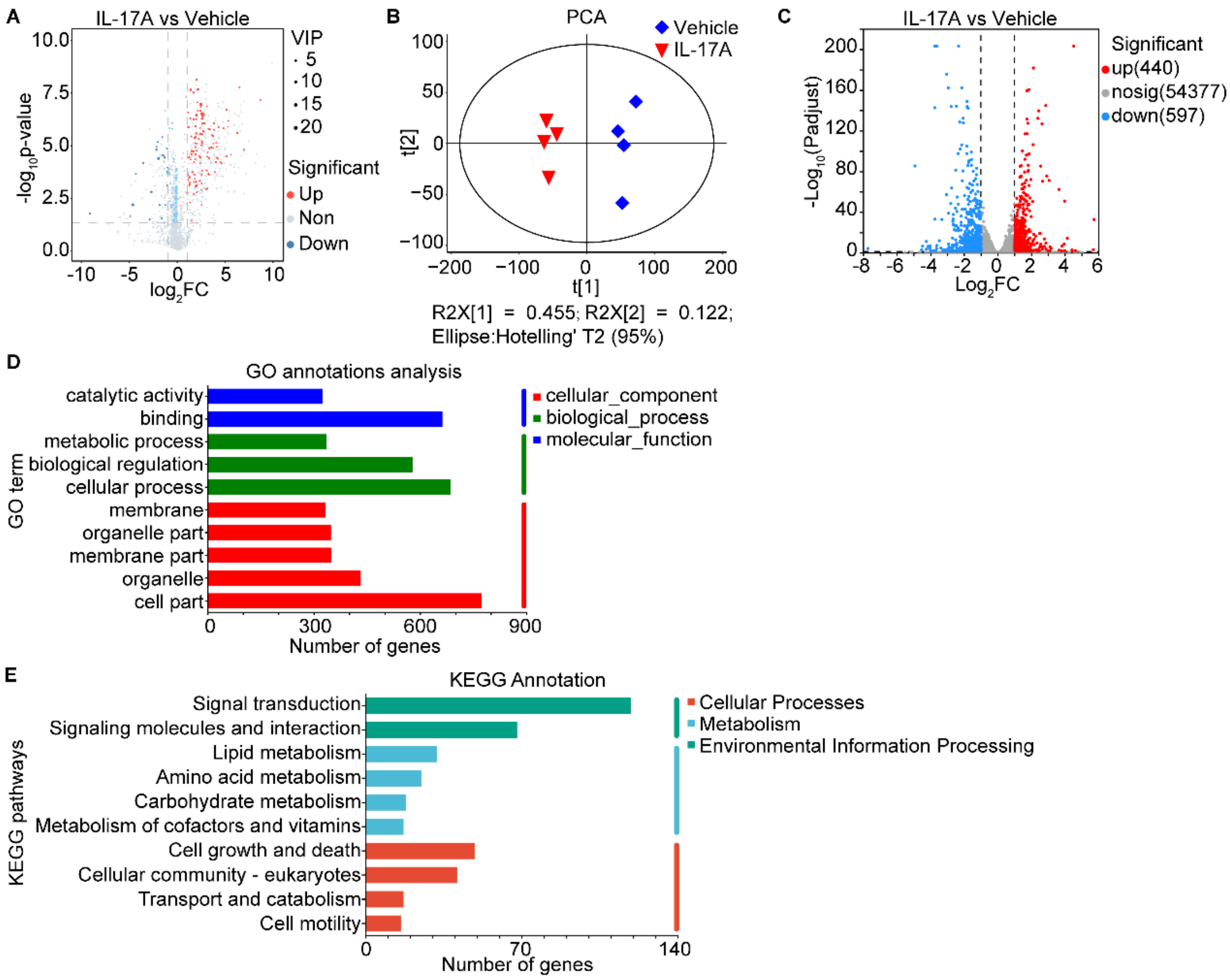

Supplementary Figure S4. Metabolic and transcriptomic profiling of primary mouse basal keratinocytes treated with IL-17A. A-B. LC-MS/MS-based metabolic profiling of primary mouse basal keratinocytes treated with IL-17A or vehicle (n = 4). Volcano plot showing significantly altered metabolites in keratinocytes treated with IL-17A versus vehicle, highlighting significantly upregulated (red) and downregulated (blue) metabolites (A). Principal component analysis (PCA) of the metabolites revealing distinct metabolic states between IL-17A- and vehicle-treated keratinocytes (B). **C-E.** Transcriptomic profiling of primary mouse basal keratinocytes treated with IL-17A or vehicle (n = 3). Volcano plot showing DEGs in keratinocytes treated with IL-17A versus vehicle, highlighting significantly upregulated (red) and downregulated (blue) genes (C). GO annotations (D) and KEGG annotations (E) analysis of DEGs between IL-17A- and vehicle-treated keratinocytes.

Supplementary Figure S5.

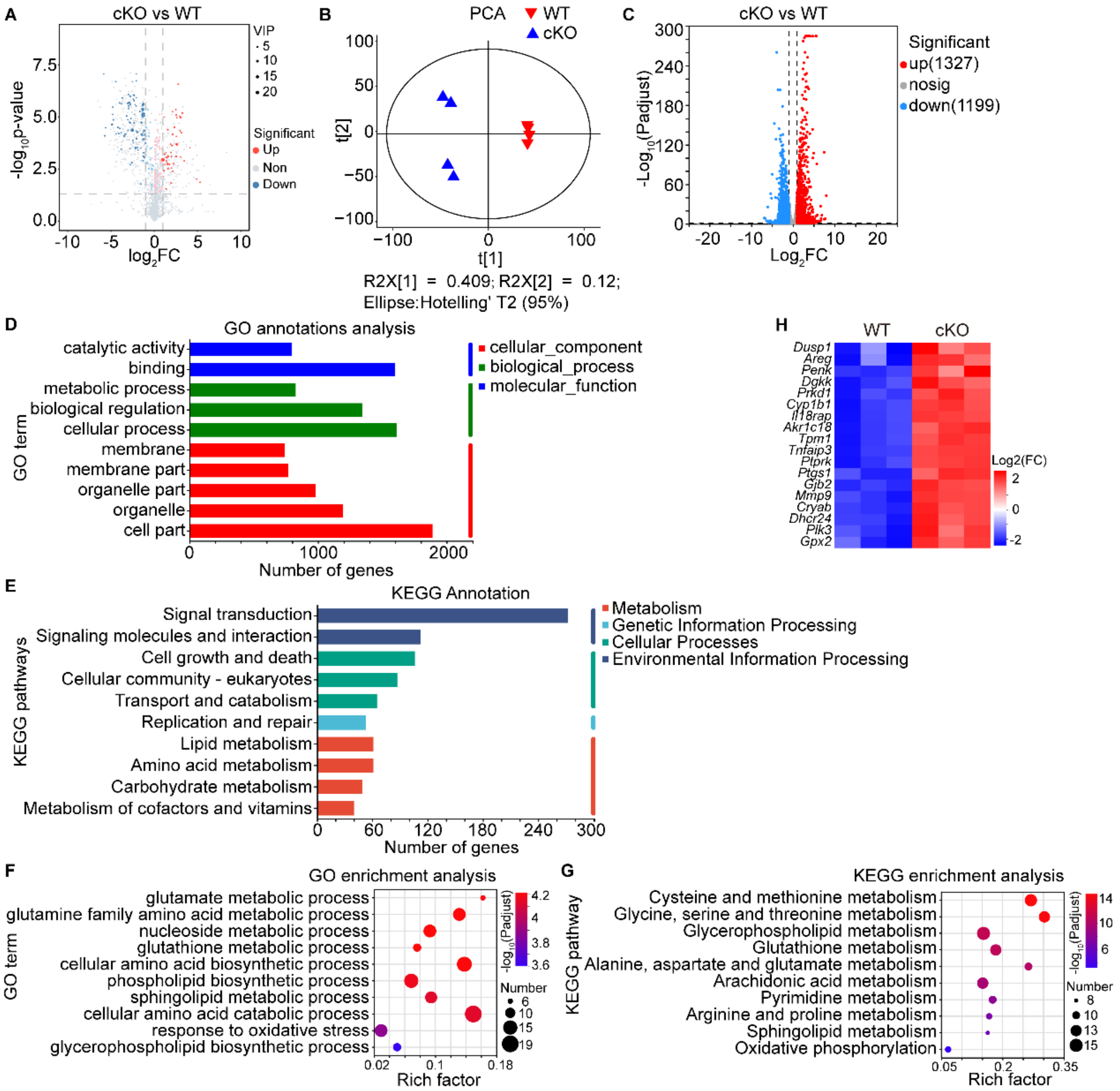

Supplementary Figure S5. Metabolic and transcriptomic profiling of IL-17A-treated primary mouse basal keratinocytes from cKO versus WT mice. A-B. LC-MS/MS-based metabolic profiling of IL-17A-treated primary basal keratinocytes isolated from WT and cKO mice (n = 4). Volcano plot showing significantly altered metabolites in keratinocytes from cKO versus WT mice, highlighting significantly upregulated (red) and downregulated (blue) metabolites (A). Principal component analysis (PCA) of the metabolites revealing distinct metabolic states between WT and cKO keratinocytes (B). **C-H.** Transcriptomic profiling of IL-17A-treated primary basal keratinocytes isolated from WT and cKO mice (n = 3). Volcano plot showing DEGs in keratinocytes from cKO versus WT mice, highlighting significantly upregulated (red) and downregulated (blue) genes (C). GO annotations (D) and KEGG annotations (E) analysis of DEGs between WT and cKO keratinocytes. Top 10 significantly enriched GO terms (F) and KEGG pathways (G) based on metabolism-related DEGs between WT and cKO keratinocytes. Heatmap of oxidative stress related genes (H) between WT and cKO keratinocytes.

Supplementary Figure S6.

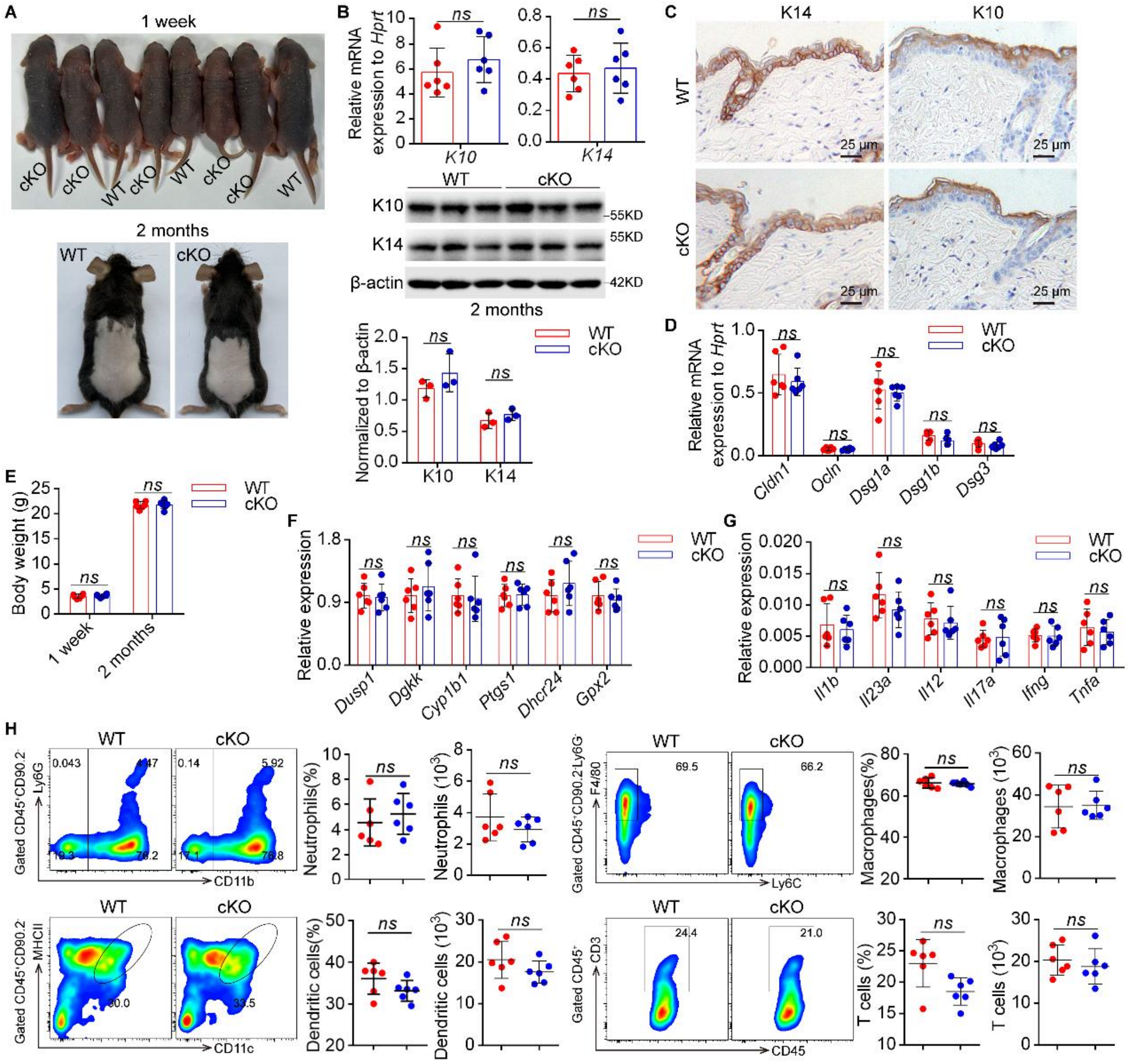

Supplementary Figure S6. Skin development and homeostasis in WT and cKO mice. A. Photos of one-week-old and depilated dorsal skin of 2-month-old WT and cKO mice reveal no discernible differences between the littermates. **B.** K10 (Keratin10) and K14 mRNA levels in harvested epidermis of 2-month-old WT (n=6) and cKO (n=6) mice (upper). K10 and K14 protein expression in the epidermis of 2-month-old WT (n=3) and cKO (n=3) (lower). **C.** Immunohistochemistry for K10 and K14 show no differences in the respective suprabasal and basal expression for the epidermal keratins between 2-month-old WT and cKO mice. **D.** RT-PCR measurement of adhesion genes in the epidermis of 2-month-old WT (n=6) and cKO (n=6) mice. **E.** Body weights of the one-week- and 2-month-old WT (n=6) and cKO (n=6) mice. **F.** RT-PCR measurement of the indicated redox genes in primary keratinocytes of 2-month-old WT (n=6) and cKO (n=6) mice. **G.** RT-PCR measurement of the indicated cytokines in skin of 2-month-old WT (n=6) and cKO (n=6) mice. **H.** Flow cytometry and statistical analysis of the percentage and number of dermal neutrophils, Macrophages, Dendritic cells and T cells of 2-month-old WT (n=6) and cKO (n=6) mice. Data are presented as mean ± SD and representative of two independent experiments with consistent results. Statistical significance was determined by two-tailed Student's t test (**B, D-H**).

Supplementary Figure S7.

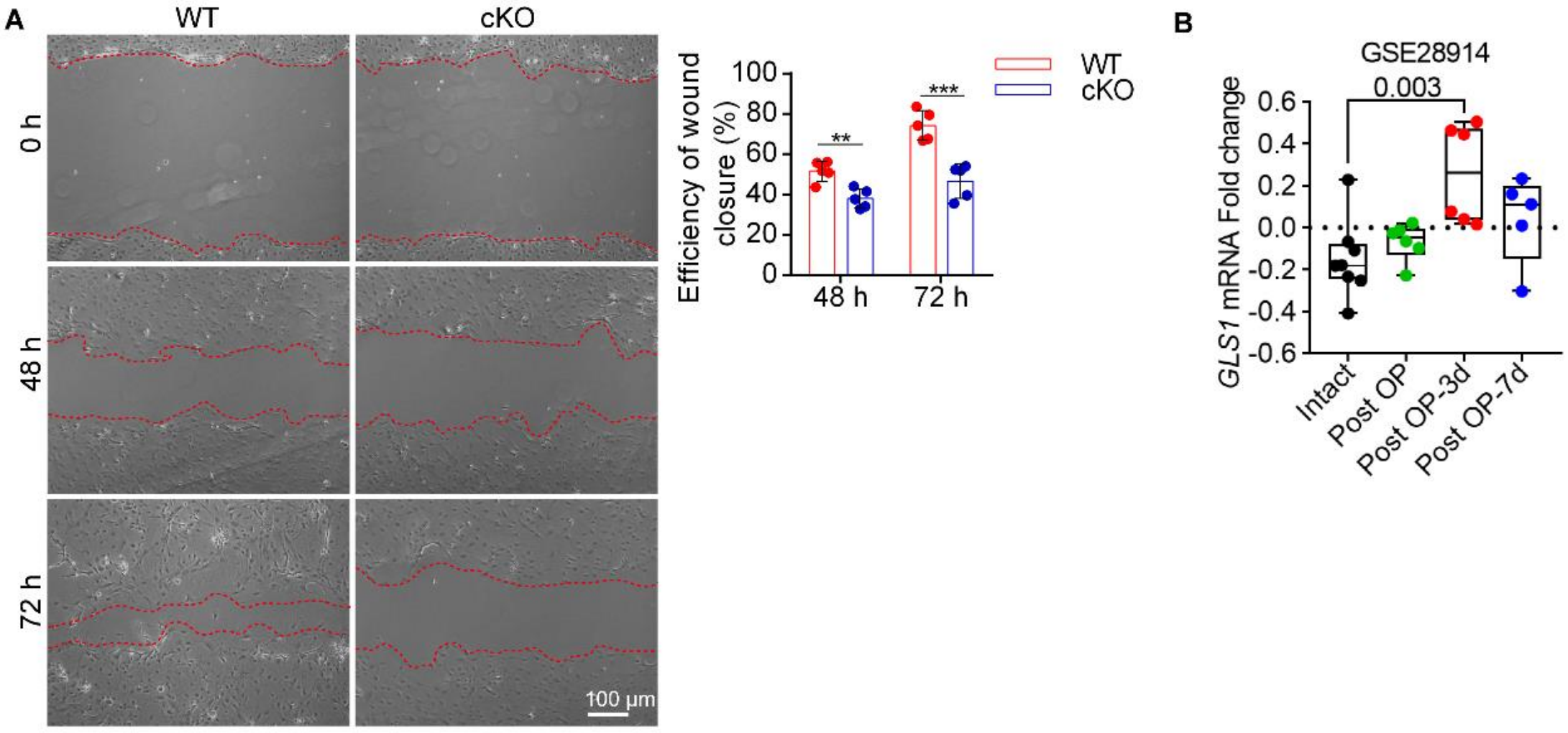

Supplementary Figure S7. Migration of primary basal keratinocytes from WT and cKO mice, and *GLS1* expression in human wound edge. A. Primary basal keratinocytes derived from WT and cKO mice were subjected to a scratch-wound assay, and wound closure was monitored at 0, 48, and 72 hours. The wound area was outlined to assess migration, and the efficiency of wound closure was quantified. (n=5). **B.** Serial biopsies from the wound edge of healthy volunteers (GSE28914) revealed significant increases in GLS1 expression 3d after wounding (n=6 individuals). Data are presented as mean ± SD and representative of three independent experiments with consistent results. Statistical significance was determined by two-tailed Student's t test (**A**), and one-way ANOVA with Tukey's multiple-comparisons test (**B**).
