# Supplementary material for: Type 3 inflammation–specific keratinocyte glutaminolysis promotes skin inflammation
Source: JCI Insight. 2026 Sep 8;11(20):e203626. doi: 10.1172/jci.insight.203626 (PMC13726564; doi:10.1172/jci.insight.203626)

Figure 2F

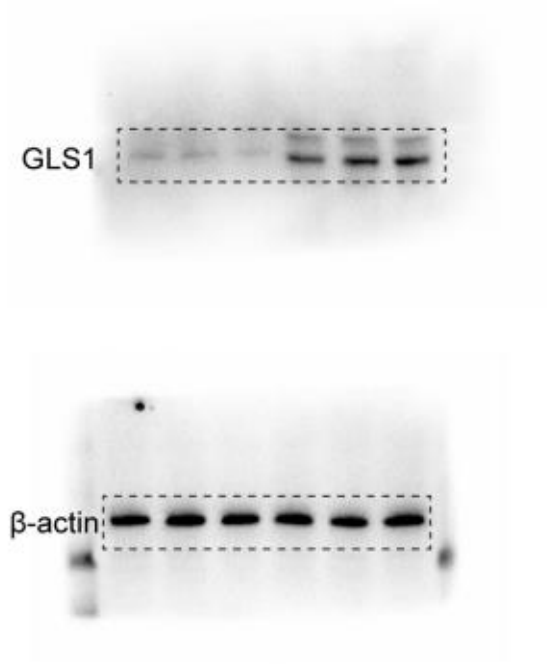

Figure 2H

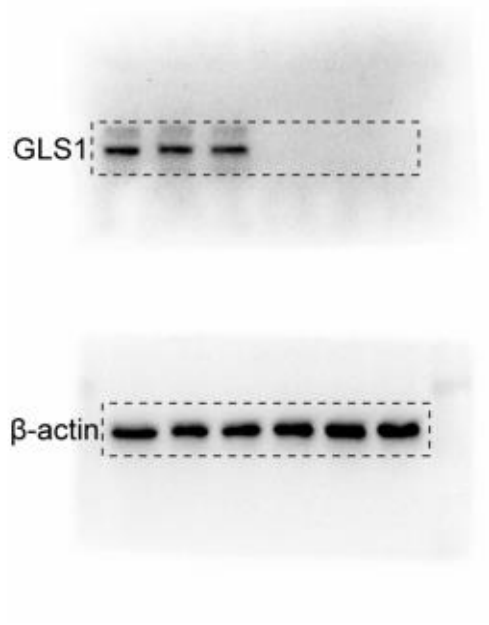

Figure 2I

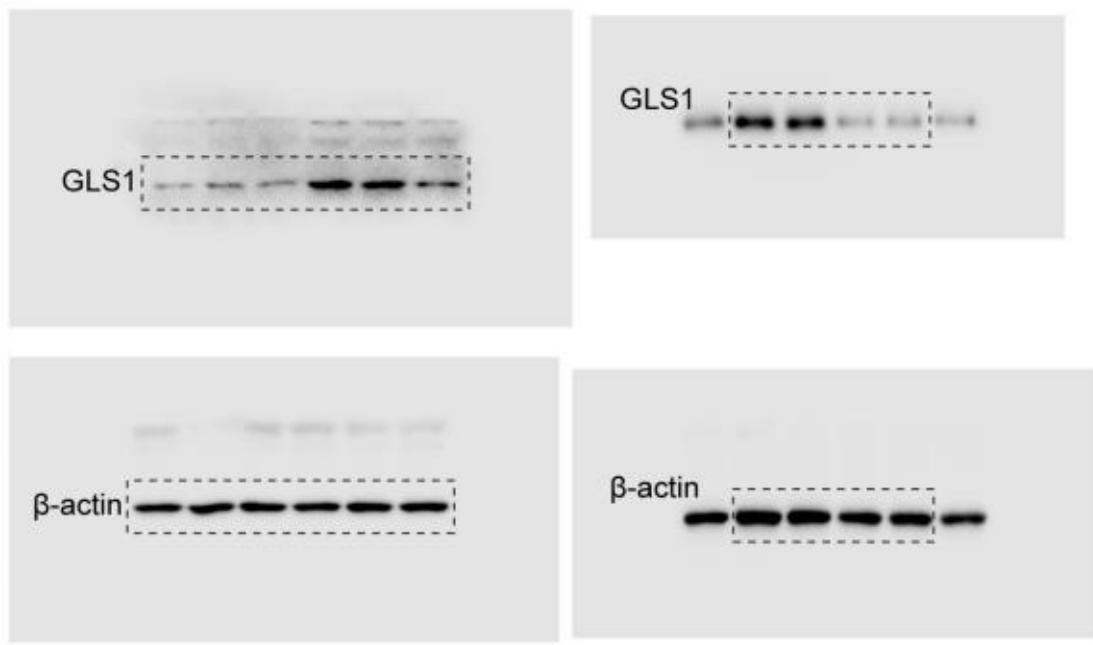

Figure 3E

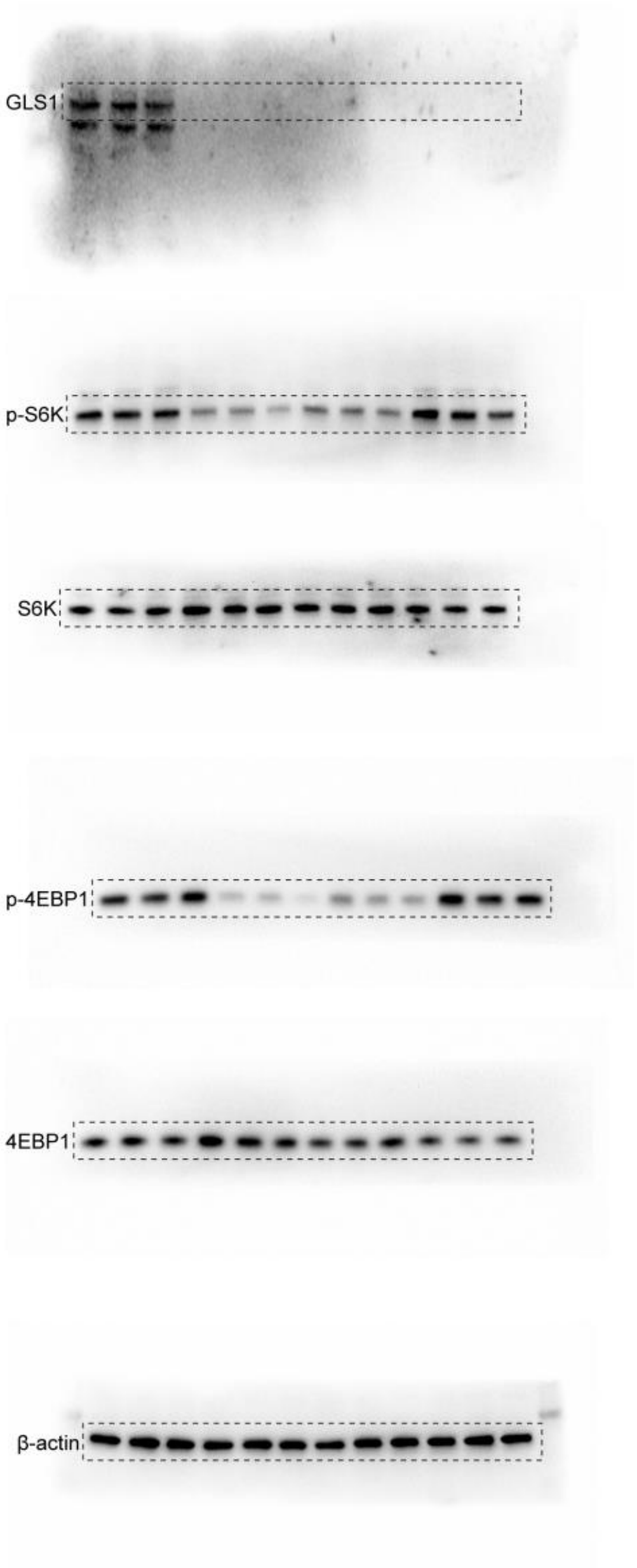

Figure 3G

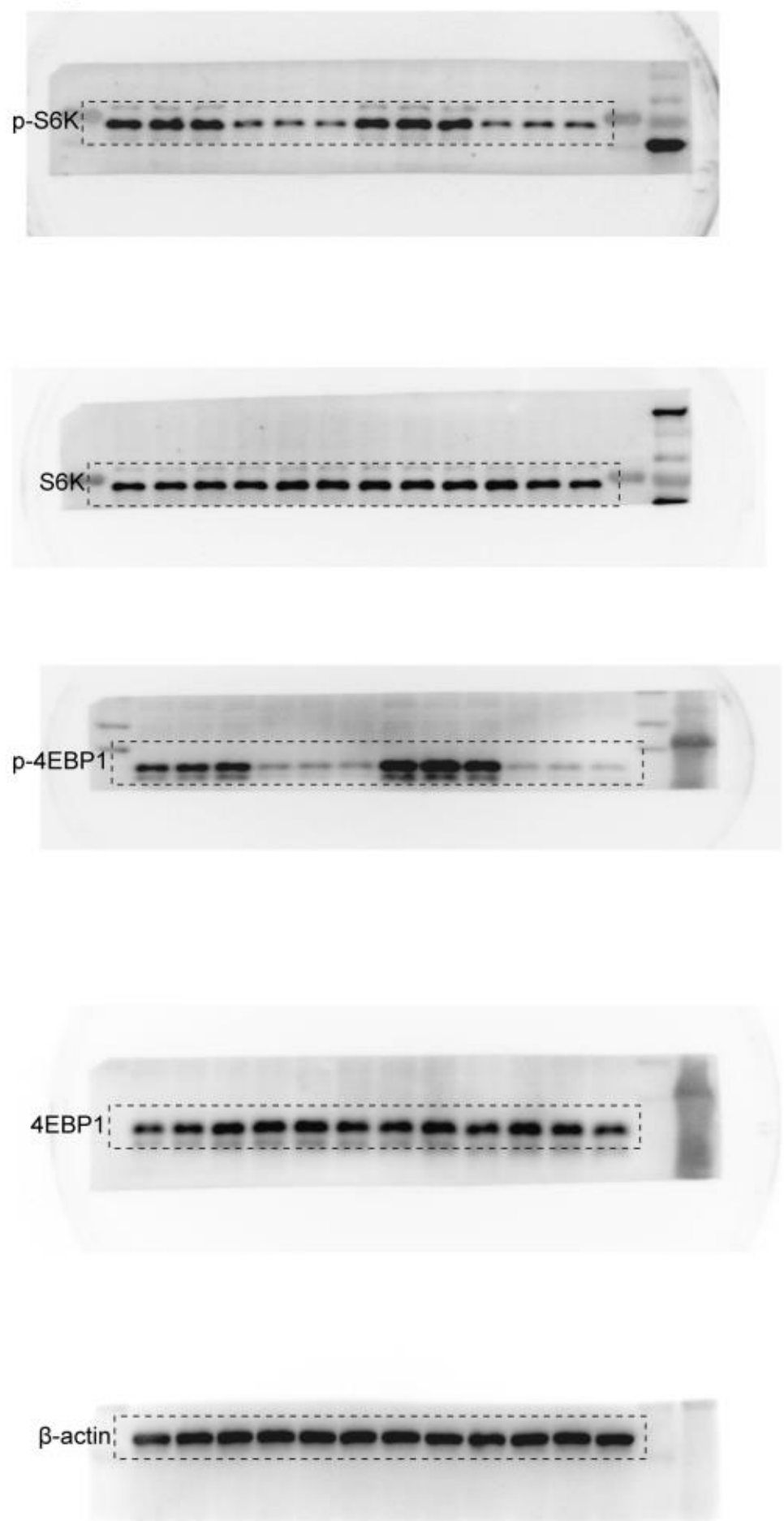

Figure 4D

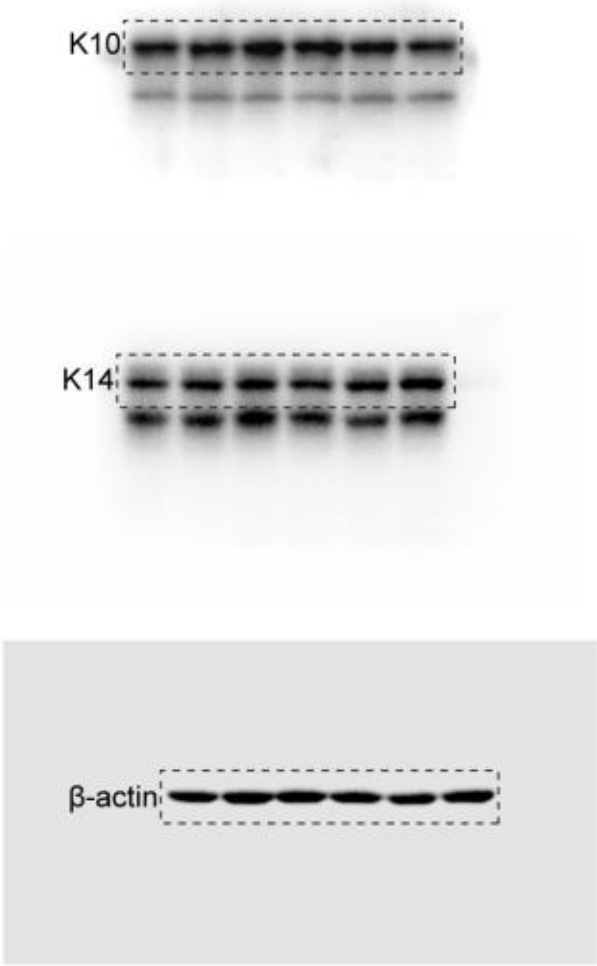

Figure 4J

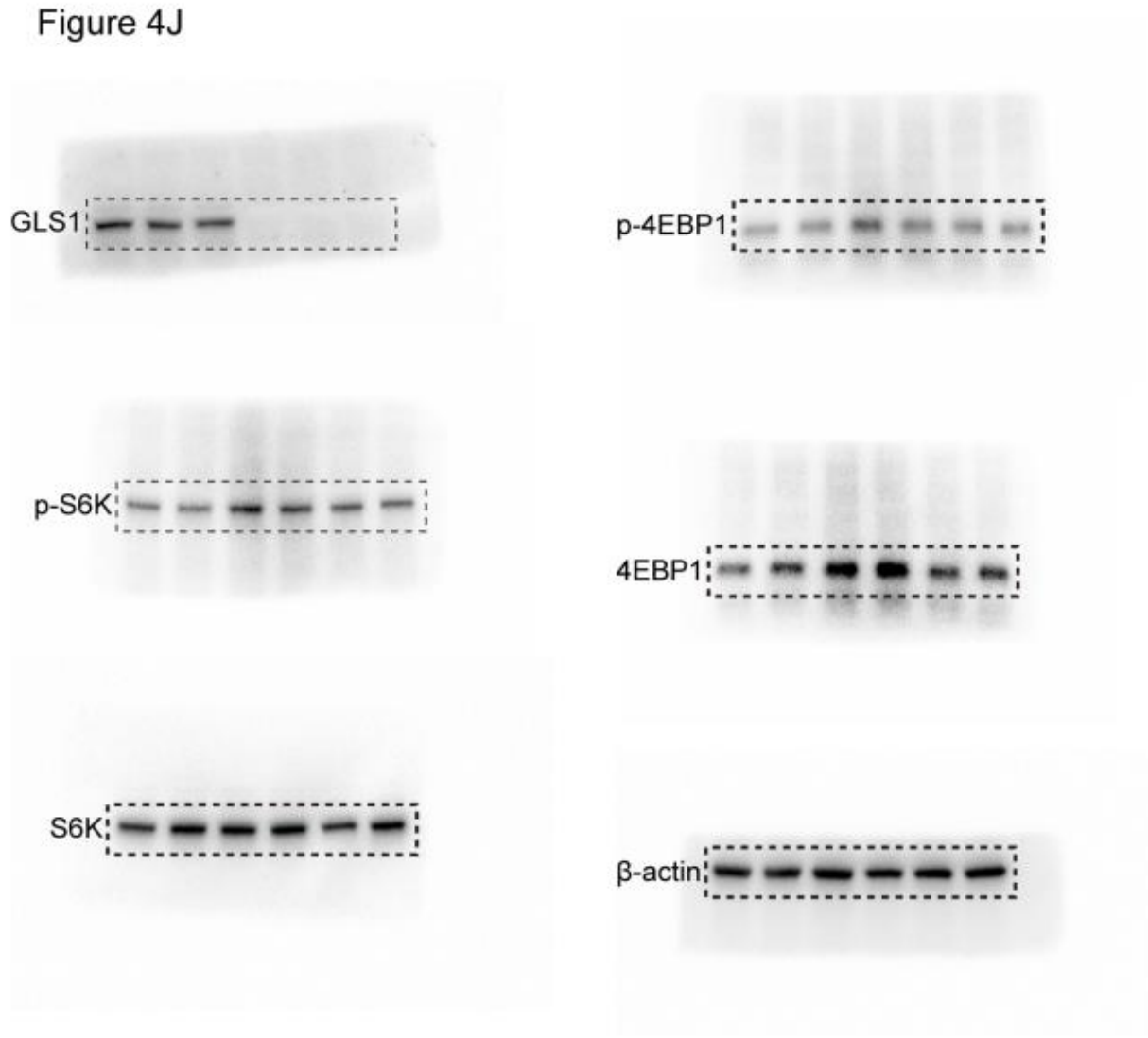

Figure 5G

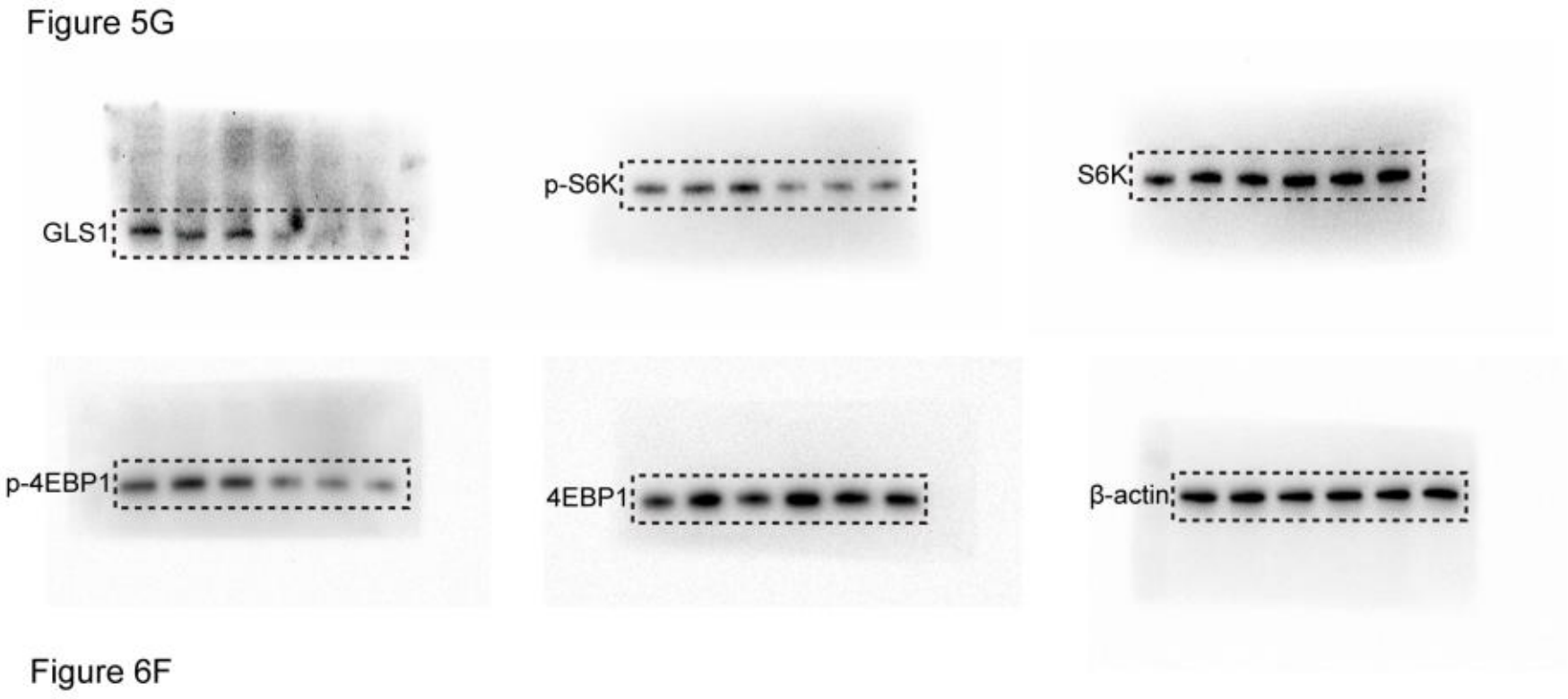

Figure 6F

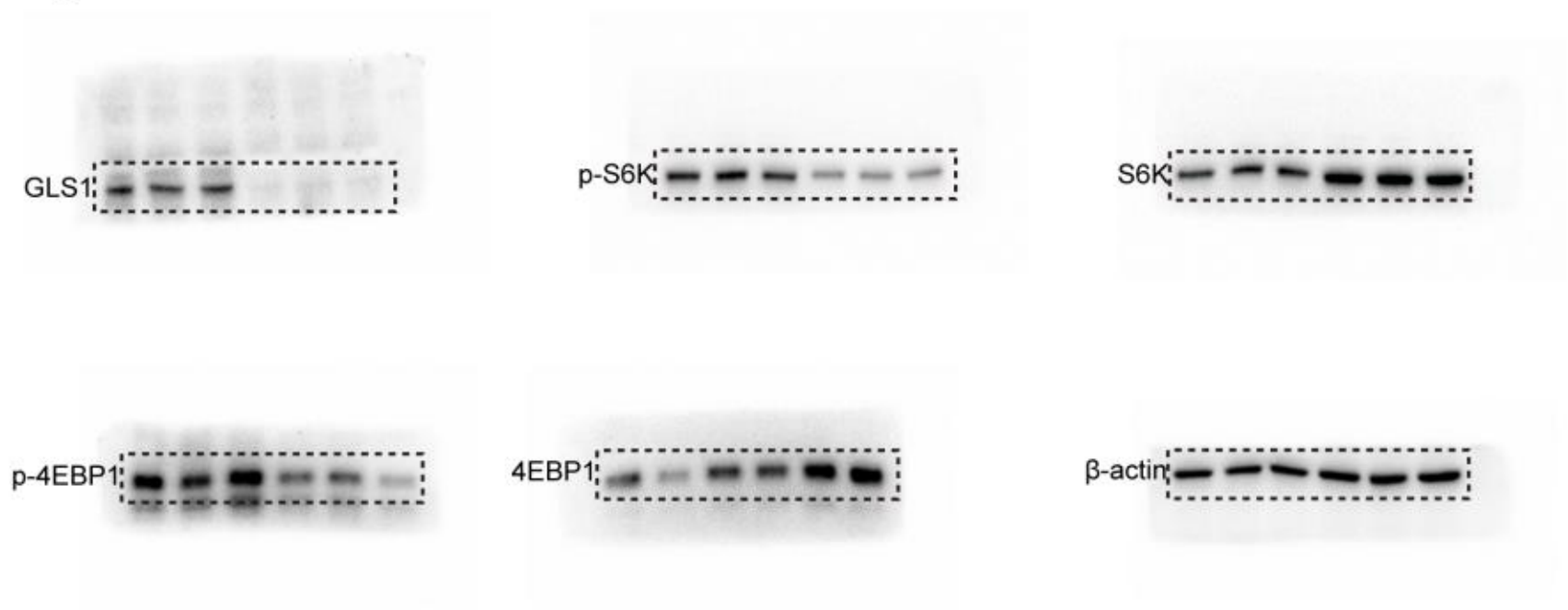

Suppl. Figure 6B

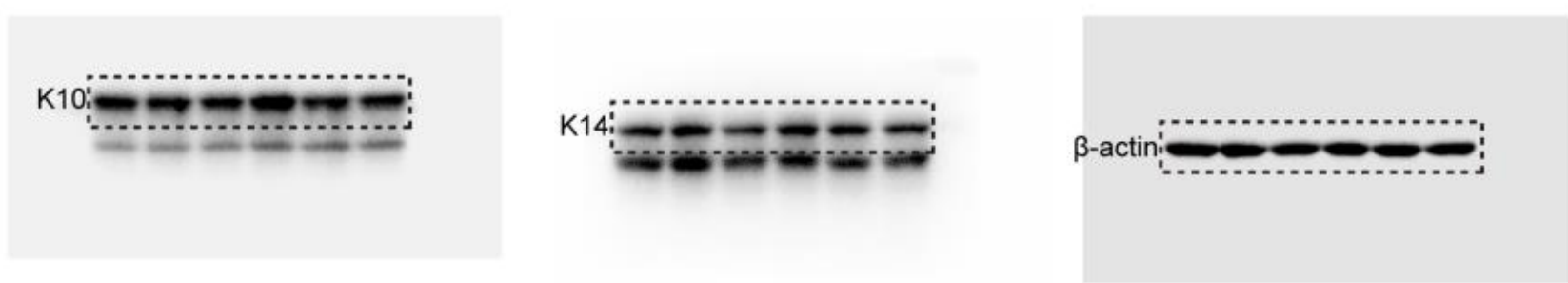

Supplement: Supplementary file 2 [file jciinsight-11-203626-s114.pdf]
